# Cognitive flexibility and N2/P3 event-related brain potentials

**DOI:** 10.1038/s41598-020-66781-5

**Published:** 2020-06-17

**Authors:** Bruno Kopp, Alexander Steinke, Antonino Visalli

**Affiliations:** Department of Neurology, Hannover Medical School, Carl-Neuberg-Straße 1, 30625 Hannover, Germany

**Keywords:** Human behaviour, Cognitive neuroscience

## Abstract

Task switching is often considered for evaluating limitations of cognitive flexibility. Switch costs are behavioural indices of limited cognitive flexibility, and switch costs may be decomposable into stimulus- and response-related fractions, as conjectured by the domain hypothesis of cognitive flexibility. According to the domain hypothesis, there exist separable stimulus- and response-related neural networks for cognitive flexibility, which should be discernible as distinct event-related potentials (ERPs). The present card-matching study allowed isolating stimulus- and response-related switch costs, while measuring ERPs evoked by task cues and target stimuli with a focus on the target-locked N2/P3 complex. Behavioural data revealed that both stimulus-task and response-task bindings contribute to switch costs. Cue-locked ERPs yielded larger anterior negativity/posterior positivity in response to switch cues compared to repeat cues. Target-locked ERPs revealed separable ERP correlates of stimulus- and response-related switch costs. P3 waveforms with fronto-central scalp distributions emerged as a corollary of stimulus-related switch costs. Fronto-centrally distributed N2 waveforms occurred when stimulus-task and response-task bindings contributed jointly to switch costs. The reported N2/P3 ERP data are commensurate with the domain hypothesis according to which there exist separable stimulus- and response-related neural networks for cognitive flexibility.

## Introduction

A better understanding of the cognitive and neural mechanisms of executive control remains a major problem for cognitive neuroscience. Executive control may be defined as maintaining behavioural goal-directedness when irrelevant information exerts a potentially interfering impact^1,2^. One source of interference may stem from slowly decaying representations of previously executed cognitive tasks^3^. The persistence of these representations may impose limitations on cognitive flexibility, a major facet of executive control, which refers to the ability to switch smoothly back and forth between different cognitive tasks^4,5^.

Cognitive flexibility is often studied in task-switching paradigms (for overview see^6–8^). In task-switching paradigms, the primary index for limitations of cognitive flexibility is switch costs^7,8^. Switch costs are usually reflected in increases in response times (RTs) and/or error rates for switch trials relative to repeat trials. On switch trials, the demanded cognitive task differs from previously executed tasks, whereas on repeat trials, the previously demanded task can simply be re-executed. One core finding is that switch costs are reduced when task cues and target stimuli are presented sufficiently long periods of time apart, rendering proactive task preparation possible to some degree^9^. However, residual switch costs remain even when cue-target intervals are very long. The occurrence of residual switch costs has been attributed to the need for reactive reconfiguration of task sets following target onset, which is due to the incompleteness of proactive task preparation when cue-target intervals end up^9^. The term task set refers to a preparatory state, which enables selecting certain types of information and ignoring other information^10^. Alternatively, residual switch costs were attributed to interfering carry-over effects from previous trials. In its simplest form, carry-over interference may emerge from residual activation of previously executed task sets. Their persistence may improve performance on repeat trials, but impair performance on switch trials^3^.

In a previous study, we employed the task-switching paradigm to test the domain hypothesis (DH) of cognitive flexibility^11^. The DH conjectures that cognitive interference between task sets arises from separate stimulus- and response-related sources. Stimulus-related sources of cognitive interference were studied in a simple card-matching paradigm that requested switching back and forth between stimulus features (i.e., colour and shape of simple geometric figures). Response-related sources of cognitive interference were studied in an even simpler card-matching paradigm that requested switching back and forth between responses (i.e., left-hand and right-hand responses to non-discriminatory stimuli). Event-related brain potentials (ERPs) served as primary outcomes, which were measured to reveal whether the neural networks for resolving stimulus- and response-related cognitive interference are separable. We found that stimulus-related cognitive interference modulated P3 waveforms with fronto-central scalp topography^12^, whereas response-related cognitive interference modulated N2 waveforms with frontal scalp topography^13^.

The ERP data that we obtained from this study were clear-cut but their explanation was not similarly clear. While separable neural networks for switching domains provided the explanation derived from the DH, task complexity was a potential moderator of these ERP data because switching between stimulus features was more difficult than switching between responses. Therefore, in a subsequent behavioural study, we employed a more sophisticated card-matching paradigm for examining stimulus- and response-related switch costs, in which we ensured that the experimental conditions were comparably complex^14^. In the following, we briefly describe the rationale behind this card-matching paradigm. It has its roots in associative binding theories of task switching^15^. According to such theories, behavioural switch costs arise from the presence of short-lived associations between mental representations of stimulus features, response properties, and task sets, as detailed below.

## Evidence for stimulus-related switch costs

Stimulus-related switch costs may originate from the presence of task-irrelevant stimulus features. These contributions are often studied by manipulating stimulus valence and congruency. While univalent stimuli present features of a single cognitive task (e.g., when executing parity/letter tasks, the stimulus ‘#3’ contains only parity-related information), bivalent stimuli comprise features for multiple tasks (e.g., ‘E3’, containing information relevant to both parity and letter tasks). In case of bivalency, stimuli may be congruent (e.g., ‘E’ and ‘3’ are both mapped to the same response) or incongruent (e.g., ‘E’ and ‘3’ are mapped to the different responses). Switch costs are usually massively reduced with univalent stimuli, whereas substantial switch costs emerge for bivalent stimuli (e.g.^16–19^,). Rogers and Monsell^18^ concluded that task-irrelevant stimulus features induce interference among task sets. In fact, despite switch cost reductions for bivalent congruent stimuli in comparison to bivalent incongruent stimuli (i.e., a congruency effect), switch costs are still larger for bivalent, congruent stimuli than for univalent stimuli (i.e., a valence effect)^20,21^. Taken together, stimulus-related switch costs are robust, well-replicable phenomena. Associative binding theories of task switching^15^, in particular associations between task-irrelevant stimulus features and task sets (i.e., stimulus-set bindings) provide a commonly accepted explanation for their occurrence^6,7^. Our card-matching paradigm allowed examining stimulus-related switch costs through manipulating the *eligibility* of stimulus features, rather than through manipulating their physical presence or absence. The term eligibility of stimulus features signifies whether or not physically present stimulus features remain viable for responding on a trial. For example, if ‘E3’ is presented as the target stimulus on a parity task, eligibility of the task-irrelevant stimulus feature ‘E’ creates a condition that emulates (congruent or incongruent) bivalency, whereas ineligibility of ‘E’ creates a condition that emulates univalency (see the Method section for details).

### Evidence for response-related switch costs

Evidence for response-related switch costs could also be established (for review see^7^). Most notably, response repetitions improve performance on repeat trials, but these benefits are often abolished or reversed on switch trials^18,22–24^. Response-repetition effects may be due to the fact that preparing a task switch also implies preparing a response switch (e.g.^22^,). Another explanation arises from the idea that responses receive inhibition to counteract perseverative tendencies^25^. The inhibitory after-effect on response-repetition trials may be compensated by task-repetition priming on the trials with simultaneous response- and task- repetitions, but that it adds to task-switch costs on response-repetition, task-switch trials (e.g.^26–30^,). Alternatively, associations between executed responses and task sets may be strengthened. Carry-over of those associations on response-repetition trials primes task repetitions, but interferes with task switches^24,31–33^. Taken together, response-repetition effects are well-replicable phenomena, although their theoretical account remains insufficiently clarified^34^ (for review see^35^). Notwithstanding these difficulties, associations between responses and task sets provide a potential mechanism that may contribute to behavioural switch costs. Our card-matching paradigm allowed examining response-related switch costs through manipulating the eligibility of responses. The term eligibility of responses signifies whether or not the previously executed responses remain viable for responding on a trial. This manipulation produces three possibilities, (1) complete ineligibility of previous responses, (2) eligibility of previous responses (but responses alternate), and (3) repetition of previous responses (see the Method section for details).

### Manipulating stimulus- and response eligibility in the card-matching paradigm

**Figure 1:**
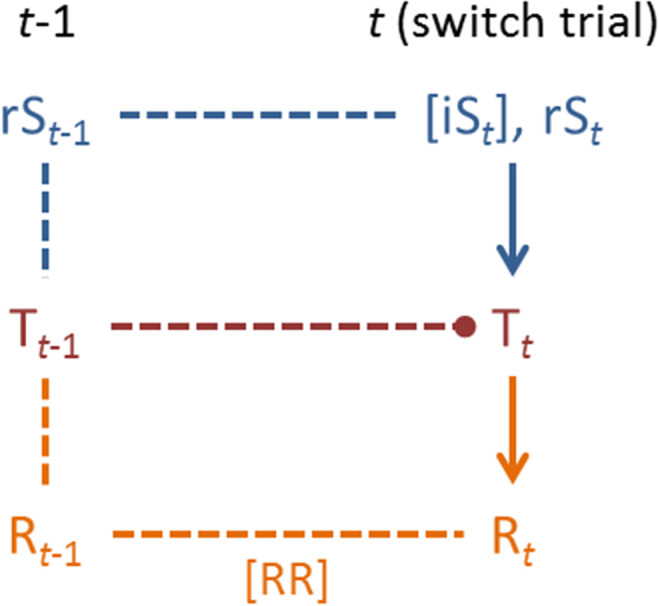
A graphical outline of the eligibility framework, which has its roots in associative binding theories of task switching^15^. Square brackets indicate manipulated variables, with [] denoting that their eligibility on trial *t* was controlled (eligible, ineligible). *t* = trial number; T = task set; rS = task-relevant stimulus feature; iS = task-irrelevant stimulus feature; R = response; RR = response repetition. *Stimulus-related switch costs:* On the depicted switch trial, T_*t*-1_ exerts carry-over interference on the execution of T_*t*_ (represented by the circular endpoint of the red dashed line) due to the reenactment (represented by the horizontal blue dashed line) of the stimulus-set binding that took place on trial *t*-1 (represented by the vertical blue dashed line). Eligible task-irrelevant stimulus features reenact stimulus-set bindings via the iS_*t*_ = rS_*t*-1_ equality, increasing carry-over interference exerted by T_*t*-1_. *Response-related switch costs:* On the depicted switch trial, T_*t*-1_ exerts carry-over interference on the execution of T_*t*_ due to the reenactment (represented by the horizontal orange dashed line) of the response-set binding that took place on trial *t*-1 (represented by the vertical orange dashed line). Response repetitions reenact response-set bindings via the R_*t*_ = R_*t*−1_ equality, increasing carry-over interference exerted by T_*t*−1_.

Manipulating the eligibility of stimulus features and responses that were relevant/executed on the previous trial may support the differentiation between stimulus-related and response-related behavioural switch costs. The eligibility framework may be most efficiently explained by presenting graphical information. Figure 1 depicts a sequence of two trials that comprises a task switch. Potential contributions from stimulus-set bindings (shown in blue), and from response-set bindings (shown in orange) to behavioural switch costs are shown. Manipulating stimulus- and response eligibility implies that stimulus features and responses on previous trials remained eligible or not on switch trials. Manipulating stimulus eligibility can be achieved by rendering the previously task-relevant stimulus feature (in-)eligible on switch trials. Hence, previously task-relevant, but meanwhile task-irrelevant stimulus features remain viable or not for responding on switch trials, thereby emulating stimulus valence. Concerning response eligibility, it can be achieved by rendering the previously executed response (in-)eligible on switch trials. Response eligibility should exert maximum effects on switch costs when previously executed responses actually need to be repeated on switch trials.

In our previous card-matching study, we employed the eligibility framework to investigate stimulus- and response-related behavioural switch costs^14^. Stimulus-related switch costs were assessed in terms of the effects of manipulating stimulus eligibility. The stimulus eligibility effects that we observed were similar to typical stimulus valence/congruency effects. Switch costs occurred solely when task-irrelevant stimulus features remained eligible on switch trials. Response-related switch costs were assessed in terms of response repetition effects. These response repetition effects were similar to those that are typically found in task-switching paradigms where strong switch costs emerge when requests for task switches and for response repetitions are combined on switch trials.

The main goal of the present study was investigating ERP correlates of stimulus- and response-related switch costs. When planning details of the study, we had four major methodological problems in mind.

First, statistical power was addressed by an apriori power analysis that rested on our previous data^14^. Second, we collected sufficiently large numbers of trials per participant and condition to ensure reliable ERP averaging^36,37^.

Third, ERP averaging faces variable degrees of trial-by-trial latency jitter, in particular when RTs are relatively slow and variable across trials (as in typical task switching paradigms). Therefore, we utilized the Residue Iteration Decomposition (RIDE) method^38^ to obtain ERP waveforms that were adjusted for trial-by-trial latency variability. The RIDE approach assumes that the ERP waveform is composed of separate components time-locked to stimulus onset (S component) and RT (R component), and other central (C) components neither locked to stimulus or RT, but having a trial-to-trial variable latency. The latency variability of these C components blurs the representation of central components in the averaged ERP, by diminishing their amplitude and altering their shape. In an iterative process, RIDE (1) estimates single trial latencies of the C components, (2) decomposes S, C, and R components based on stimulus onsets, estimated C latencies, and RTs, (3) uses the decomposed C to re-estimate C latencies, (4) repeats steps 2 and 3 until convergence. The RIDE algorithm then returns a reconstructed ERP compensating for latency variability in the C and R components.

Finally, we wanted to avoid circular statistical analyses of the ERP waveforms^39,40^. Circularity occurs when the selection of the details for inferential data analysis relies on an advance inspection of the ERP waveforms that are being analysed. We utilized a mass univariate approach^41,42^ to the analysis of ERP differences between experimental conditions.

The DH predicts separable ERP correlates of stimulus- and response-related switch costs. Based on our previous ERP findings^11^, stimulus-related switch costs should be associated with the occurrence of fronto-centrally distributed P3 waveforms, while response-related switch costs should be associated with the occurrence of frontally distributed N2 waveforms.

## Method

### Participants

Forty undergraduate psychology students (36 female; mean age = 21.8 yrs, *SD* = 3.2 yrs) participated for course credit. All participants had normal or corrected-to-normal vision. Four participants had to be excluded from analyses because they were outliers in the number of trials with RT > 2000 (outliers were identified using the MATLAB function *isoutlier* with “quartiles” as specified method, which considers outliers the elements of an array A more that 1.5 interquartile ranges above the upper quartile or below the lower quartile of A^43^), resulting in a final sample of *N* = 36 (32 female; mean age = 21.6 yrs, *SD* = 3.1 yrs). Note that these exclusion criteria are different from those that we applied in our previous study^14^. In that study, we did not exclude any subjects, but rather all trials slower than three *SD* above individual mean RTs. Here, we altered our analysis strategy because we prioritized consistency between behavioural and EEG data analyses. The named alteration of the behavioural data analysis did not lead to any noticeable changes in the results. Data collection was approved by the local ethics committee at the Department of Psychology of the Technische Universität Braunschweig (DM-2016-09). All participants gave informed consent in accordance with the Declaration of Helsinki.

An effect size estimate for latency switch costs on the Cued Card Matching Task (CCMT) was taken from our previous behavioural study^14^. The highest order interaction found there was associated with an effect size of Cohen’s *f* = 0.77. The current study was designed to have sufficient statistical power (i.e., 1-*β* = 0.95) to find an effect of this size, given *α* = 0.05. G-Power^44^ indicated that a minimum sample size of *N* ≥ 26 was required for that purpose. However, we wanted to collect data from a somewhat larger sample since - in addition to analysing the replicability of our previous behavioural findings^14^ - we were interested in analysing putatively more subtle differences between ERP measures across conditions.

### The cued card matching task

**Figure 2:**
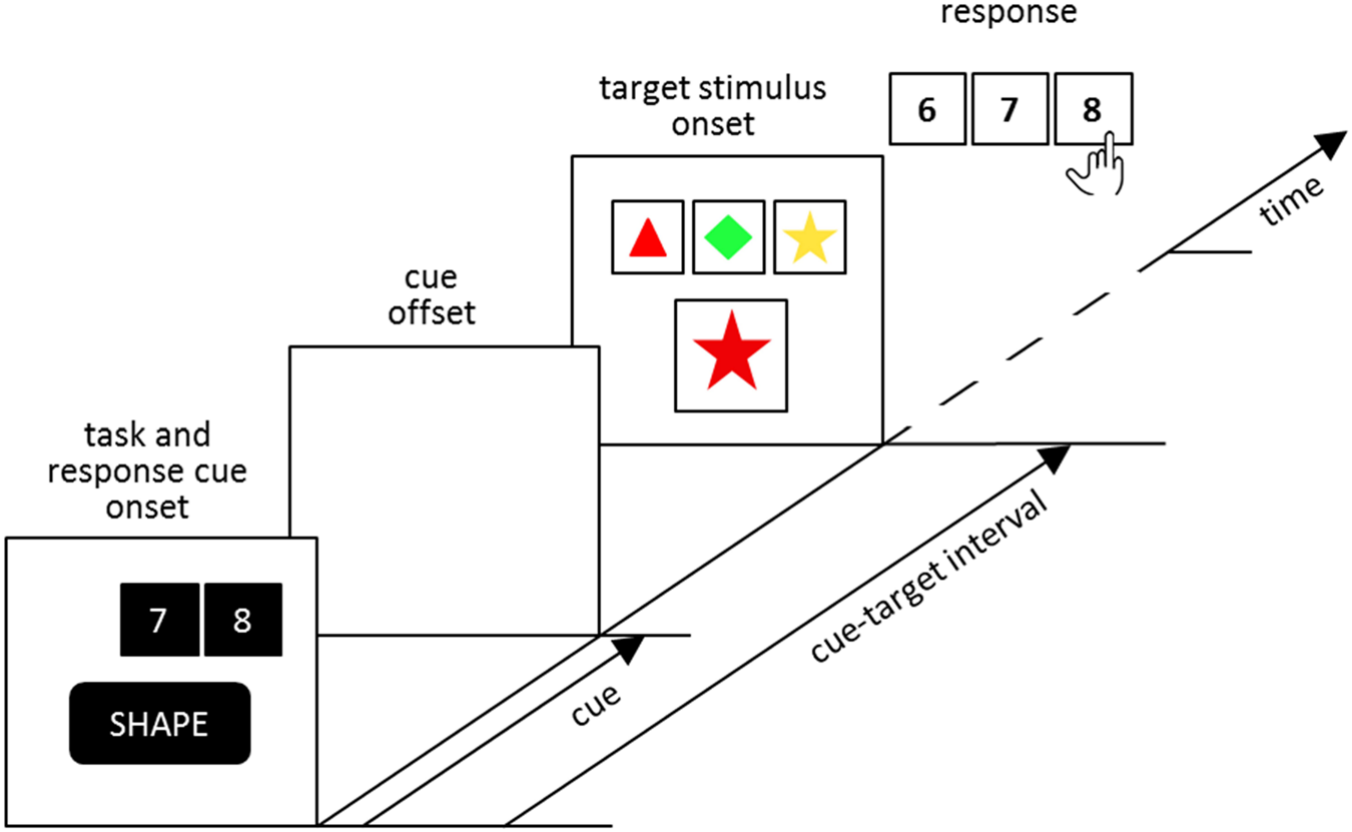
An exemplified trial on the Cued Card Matching Task (CCMT). Response and task cues were followed by targets. Three reference cards and one response card constituted the target, and both types of cards depicted coloured geometrical shapes. The task required matching the response card against the three horizontally aligned reference cards. On each trial, two tasks were possible: colour or shape. In our example trial, the red star matches the red triangle (identical with regard to colour), and it matches the yellow star (identical with regard to shape), but there is no match between the red star and the green diamond (hence the middle reference card is not viable for responding). The correct response to the cued task SHAPE is STAR, to be indicated by choosing the right reference card. The response to the irrelevant (competitor) task COLOR would be RED, to-be-indicated by choosing the left reference card. The two response cues 7 and 8 render both the middle (non-viable) and the right (shape task) reference card/response eligible. The omitted response cue 6 renders the left (colour task) reference card/response ineligible. Hence, the depicted trial exemplifies the *in*eligible competitor-task condition. Further study details can be learned from watching the task video, which is available in the Open Science Framework repository (https://www.osf.io/cqkhn/).

The CCMT was designed using OpenSesame^45^ and displayed on a 24 inch flat screen. As shown in Fig. 2, on each trial, participants matched a response card (5.6 × 5.6 cm) to one of three horizontally aligned reference cards (3.9 × 3.9 cm each). Response cards and reference cards together constituted the target-stimulus configuration. The entire target-stimulus configuration measured 12.4 cm horizontally and 10.8 cm vertically. Response cards were presented centrally, whereas the reference cards appeared 6 cm above the centre of the screen. Viewing distance amounted to 120 cm. Responses were collected using a Cedrus response pad (RB830).

Matching response cards (yellow diamond, yellow triangle, red diamond, red star, green triangle, or green star) and reference cards (red triangle, green diamond, and yellow star) was possible according to two distinct sorting rules, i.e., their shape or colour. We eliminated all potential response cards that were identical to a reference card (i.e., cards that depict a red triangle, a green diamond, and a yellow star). Using task-switching jargon, all response cards were bivalent and incongruent, such that they afforded responses to two different reference cards on each trial. At the same time, this feature of the CCMT implies that one of the three reference cards remained infeasible for responding (see Fig. 2).

Responses were based on comparisons between the relevant response-card feature and the corresponding reference-card feature. In the example that is depicted in Fig. 2, the task cue requested matching cards according to SHAPE. The reference card showing a STAR was the correct choice since here the response card also showed a STAR. Participants indicated their choice by pressing one of three adjacent keys on the response pad (with the index, middle, and ring finger of their right hand, respectively)^14^. The alignment of the three response keys (6, 7, 8) mapped the spatial positions of the three reference cards on the screen (on the left, red triangle = key 6, in the centre green diamond = key 7, on the right yellow star = key 8). Target stimuli remained on the screen until a response was recorded. Time intervals between responses and onset of the next trial were held constant at 1.000 ms. Erroneous responses were followed by a 400 Hz feedback tone presented for 100 ms.

Cue onsets preceded target onsets by 1.100 ms. Cues and target stimuli were presented sufficiently long periods of time apart in order to render proactive task preparation possible to some degree, thereby focusing ourselves on an analysis of residual switch costs in behavioural measures^9^. At the same time, this CCMT feature gave us the opportunity to study ERP correlates of proactive task preparation, which might happen during the course of these long cue-target intervals.

The cues consisted of two components: First, the relevant task was announced by written task cues (i.e., either the German word for shape [FORM] or for colour [FARBE]). Second, two response cues were presented simultaneously with the task cue. Response cues consisted of two black squares matched in size to reference-card size. The spatial position of the two visible squares indicated those two reference cards that remained eligible on the current trial, whereas the omitted third square indicated the reference card that turned ineligible on that trial. To further facilitate processing of the response cues, the corresponding response keys (i.e., characters 6, 7, or 8) were written in white capital characters (Calibri, bold, 60 pts) against the black background of the squares (e.g., the left response cue contained character 6). As in our previous study^14^, cues were only transiently presented (the duration of the presentation on screen for both, task and response cues, amounted to 600 ms), and cue offset was followed by 500 ms of white screen until target onset (see Fig. 2).

### The factorial design

**Figure 3:**
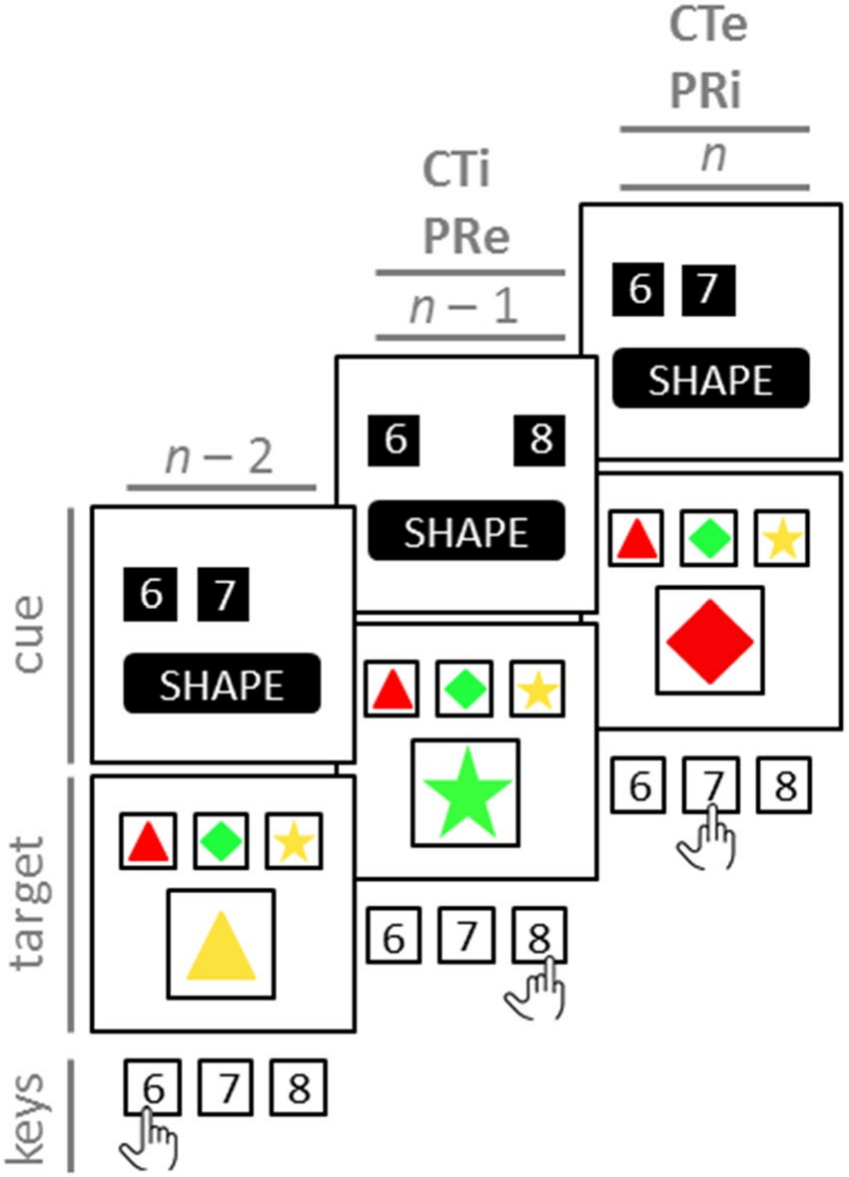
Three exemplified consecutive trials on the Cued Card Matching Task (CCMT). Two tasks are possible on each trial, that is, colour and shape. Trial *n* shows an “eligible competitor task” (CTe), “ineligible previous response” (PRi) trial. Here, the correct response is DIAMOND, hence the middle reference card (7 key). Given the two response cues on trial *n*, the irrelevant task (colour) remains eligible (RED, left response card). In contrast, the previous response (i.e., the 8 key on trial *n* – 1) is made ineligible by the response cues. Trial *n* – 1 shows an “ineligible competitor task” (CTi), “eligible previous response” (PRe) trial. Here, the correct response is STAR, hence the right reference card (8 key). Given the two response cues on trial *n* – 1, the irrelevant task (colour) is made ineligible (GREEN, middle response card) by the response cues. In contrast, the previous response remains eligible (i.e., the 6 key on trial *n* – 2). CTi = competitor task ineligible; CTe = competitor task eligible; PRe = previous response eligible; PRi = previous response ineligible.

Introducing response cues allowed examining how competitor-task eligibility (CTE) affected switch costs^14^, as shown in Fig. 3. The competitor task remained *eligible* when the two response cues rendered both viable reference cards eligible (see as an example trial *n* of Fig. 3). The competitor task turned *ineligible* when the two response cues rendered the irrelevant reference card ineligible (see as examples Fig. 2 and trials *n* – 2 and *n* – 1 of Fig. 3). When competitor tasks remained eligible, response cards retained their bivalent and incongruent nature, that is, they afforded two different reference cards. Response cards emulated univalent targets when competitor tasks were ineligible, that is, when response cards did not afford the irrelevant reference card. We refer to this experimental manipulation as CTE, with competitor task eligible (CTe) and competitor task ineligible (CTi) as the two factor levels.

Response cuing also provided novel opportunities to analyse the effects of trial-by-trial response variability on switch costs. Assuming trial-by-trial response alternations (the case of response repetitions will be discussed below), there are two possibilities: First, as illustrated in Fig. 3, the omitted response cue on trial *n* rendered the previous response on trial *n* – 1 ineligible. Second, the omitted response cue on trial *n* – 1 left the previous response on trial *n* – 2 eligible. Response cards thereby emulated univalent and bivalent responses, respectively. They emulated univalent responses when they afforded only those responses that were not executed on the preceding trial, which is equivalent to the ineligibility of the previously executed response. When the previously executed response remained eligible, they emulated bivalent responses. We refer to this experimental manipulation as previous response eligibility (PRE), with previous response ineligible (PRi) and previous response eligible (PRe) as factor levels.

The introduction of response cues made it possible to manipulate CTE and PRE on a trial-by-trial basis in a factorial design. Taken together, the present study employed a 2 (Task Sequence: repeat vs. switch trial) × 2 (CTE: CTi vs. CTe) × 2 (PRE: PRi vs. PRe) × 2 (Response Sequence: repetition vs. alternation), with Response Sequence being nested in PRE because response repetition (factor level of Response Sequence) was impossible when the previous response was ineligible. For omnibus analyses across all factor levels of our experimental design, we introduced the nested factor Response Variability (PRi | response alternation, PRe | response alternation, PRe | response repetition).

### Procedural details

Ninety trials were administered in each of these twelve conditions, resulting in 1.080 trials. Pseudorandomized sequences of trials were created to ascertain that each of the conditions consisted in the desired ninety trials. Neither response cues nor target recurred on two consecutive trials. The trials were presented in six blocks of 180 trials each. Prior to each block, 10 warm-up trials were administered.

Participants were instructed to pay attention to both the task cues and the response cues. Prior to the experimental sequence, participants’ understanding of the task was ascertained by running 36 practice trials. The experiment was run in a quiet and comfortably illuminated room, and lasted about 150 minutes per participant.

### EEG data acquisition

Continuous EEG was recorded with a BrainAmp amplifier from 30 Ag-AgCl electrodes (Brain Products, Gilching, Germany) using BrainVision Recorder 1.2 (Brain Products, Gilching, Germany) at a sampling rate of 250 Hz with a bandpass filter of 0.01–70 Hz. Impedance was kept below 10 kΩ throughout the recording. Electrodes were mounted on an actiCap (EASYCAP, Herrsching, Germany) according to the international 10–20 system. Two additional electrodes were placed at the suborbital ridge (vEOG) and external ocular canthus (hEOG) of the right eye to control for ocular artefacts. FCz was used as reference electrode.

### Data analysis

#### Behavioural data

We analysed mean RTs on correct trials for each individual. Therefore, we excluded trials slower than 2.000 ms, erroneous trials, and post-error trials (see Supplementary Table S1). Post-error trials were excluded for two reasons: (i) first, to control for effects of post-error slowing^46^; (ii) second, because the sequential analyses (Task Sequence, CTE, PRE, and Response Sequence) relied on correct responding on the previous trial. Percent errors (PE) are also considered to check for potential speed-accuracy trade-offs. For analysis of PE, we excluded all trials slower than 2.000 ms, post-error trials, as well as rarely occurring “odd” error trials (i.e., when the non-viable reference card was chosen, 0.2% of all trials). As in our previous study^14^, the linear integrated speed-accuracy score (LISAS)^47^ was computed. LISAS represents an integrated measure that is based on a linear combination of RTs and PE (in arbitrary units, a.u.). Throughout this manuscript, the term ‘integrated switch costs’ refers to LISAS, which integrate switch costs in terms of RTs and PE. Vandierendonck^47^ recommended refraining from calculating the LISAS when RT and PE are negatively correlated, but this was not the case in any of our conditions (cf. Supplementary Table S2).

We commenced statistical inference by a sequence of repeated measures ANOVAs with RTs, PE, and LISAS as dependent variables. First, we conducted an omnibus repeated measures ANOVA including the variables Task Sequence (repeat, switch), CTE (ineligible, eligible), and Response Variability (PRi | response alternation, PRe | response alternation, PRe | response repetition). Follow-up analyses comprised 1) a repeated measures ANOVA focusing exclusively on PRi trials, which included the factors Task Sequence (repeat, switch) and CTE (ineligible, eligible), 2) a repeated measures ANOVA focusing exclusively on response alternation trials, which included the variables Task Sequence (repeat, switch), CTE (ineligible, eligible), and PRE (ineligible, eligible), and 3) a repeated measures ANOVA focusing exclusively on PRe trials, which included the variables Task Sequence (repeat, switch), CTE (ineligible, eligible), and Response Sequence (alternation, repetition). As a control analysis, we also run a linear mixed-model on LISAS, which can handle unbalanced design with nested factors. This analysis included as fixed terms Task Sequence, CTE, PRE, Response Sequence and all possible interactions. The results, which are in line with our main results, are presented in the Supplementary Table S3.

Statistical significance was determined at *α* = 0.05. Greenhouse-Geisser corrected *p*-values are reported for all main effects and interactions that involve factors with more than two levels, but original degrees of freedom are reported. Behavioural data was analysed by means of R version 3.4.2^48^ and SPSS 25.

#### EEG data

EEG Data was analysed using MATLAB R2018a.

#### EEG pre-processing

For each participant, offline processing of EEG signal was performed using custom MATLAB scripts, which included functions from the EEGLAB environment (version 14.1.2b)^49^, the FastICA algorithm^50^, and the RIDE toolbox^38^.

As a preliminary step for the ICA decomposition - used for EEG artefact removal - continuous EEG data were band-pass filtered using a one-pass non-casual zero-phase Kaiser windowed sinc FIR filter (cut-off frequencies = 2 and 40 Hz; transition bandwidth = 4 and 20 Hz for the high- and low-pass filters, respectively; passband ripple = 0.001). The *clean_rawdata* EEGLAB function was used to remove noisy channels (channel criterion = 0.8) and short-time burst (burst criterion = 20 *SD*). A maximum of 3 channels per subject (mean = 0.9, *SD* = 1) were removed. Finally, the FastICA algorithm^50^ was employed to obtain ICA weights and sphering matrices.

The 2-Hz high-pass filter was applied to remove low-frequency drifts in order to improve the ICA solution^51^. However the use of such an extreme high-pass filter cut-off may attenuate ERP effects and introduce distortions^52^. For this reason, the ICA solution calculated on 2-Hz high-pass filtered data was then applied on continuous EEG data band-pass filtered using a one-pass non-causal zero-phase Kaiser windowed sinc FIR filter with cut-off frequencies of 0.1 (transition bandwidth = 0.2) and 40 Hz (transition bandwidth = 20) for the high- and low-pass filter, respectively. Indeed, the 0.1-Hz cut-off frequency seems a good trade-off between waveform distortions and statistical power^52^. Before applying the ICA solution, noisy channels identified in 2-Hz high-pass filtered data were removed. Subsequently, the EEGLAB extension SASICA^53^ was used to guide the identification and exclusion of artifact independent components (e.g., blinks, eye movements, muscle activity, misconnected channels). Removed channels were interpolated using spherical splines^54^, and continuous EEG data were re-referenced to the average of all EEG electrodes adding FCz channel back to the data.

For the target-locked analysis, data were segmented into epochs [−50, 2.300 ms] with respect to target onset. Error and post-error trials were excluded from the subsequent analysis, as well as trials with response times (RTs) slower than 2.000 ms (the 2.300 ms upper limit for epoch segmentation allows to perform the below descripted RIDE procedure with a time window of [−300, 300 ms] around RT latency). Then, we performed automatic detection and rejection of artifactual and/or outlier epochs based on extreme values, linear trend, improbability, and kurtosis^55^. Rejection thresholds were: +/−75 µV for the extreme values identification; slope exceeding 50 µV (R-square limit = 0.2) for the linear trend test; 7 *SD* and (for each channel) and 3 *SD* (for all channels) for both improbability and kurtosis tests. Furthermore, epochs were visually inspected and any epoch containing residual artifacts were manually rejected (number of excluded and survived epochs per condition are presented in Supplementary Tables S1 and S4). The remaining epochs were baseline-corrected by removing the mean voltage calculated over the time window [−50, 50] with respect to target onset, in order to minimize potential misalignments of the waveform based on anticipatory neural activity, such as CNV^56^. Epochs were pooled separately for each of the 12 trial types, and the RIDE toolbox^38^ was employed to obtain reconstructed ERP waveforms adjusted for trial-to-trial latency variability in central and response components.

Visual inspection of conventional grand-averaged ERPs showed the presence of 4 central components (P2-like, two N2-like, and a later slow-wave; see Supplementary Material). Therefore, the RIDE analysis was run using one S component (stimulus-locked time-window function in ms for waveform extraction: [0, 300]), four C components (stimulus-locked time windows in ms: [100, 400], [200, 600], [300, 700], [400, 1.000]), and R (RT-locked windows in ms: [−300, 300]).

For the cue-locked analysis, data were segmented into epochs starting 100 ms before cue onset and lasting 1.300 ms after cue onset (i.e. 200 ms after target onset in order to included target onset in the RIDE analyses and subsequently test differences in the baseline time window used in the target-locked analyses). Error and post-error trials were excluded from the subsequent analysis. Outlier and/or artifactual epochs were identified and discarded using the same procedure employed in the target-locked analyses (number of excluded and survived epochs per condition are presented in Supplementary Tables S1 and S5). The remaining epochs were baseline-corrected by removing the mean voltage calculated over the time window [−100, 0] with respect to cue onset and pooled separately for each of the twelve trial types. Reconstructed ERPs compensating for C latency variability were obtained using RIDE, which separated three S components for cue onset, cue offset, and target onset (time windows in ms with respect to target cue were, respectively: [0, 300], [600, 900], [1.100, 1.200]) and one C component (time window in ms: [300, 1.300]).

#### EEG inferential statistics

The *ept-TFCE* toolbox^41^ was used for mass univariate analysis of ERP differences between experimental conditions. This toolbox makes use of a Threshold-Free Cluster-Enhancement (TFCE) method^42^ in conjunction with permutation-based statistics (5.000 was the number of permutations used in all our tests, *α* = 0.05).

Concerning the target-locked analysis, TFCE analyses were performed in the time window [0 1.000 ms] after target onset. Differently from behavioural analyses, we were not able to run an omnibus repeated measures ANOVA including the variables Task Sequence, CTE, and Response Variability since the ept_TFCE toolbox is compatible with ANOVA models including only two factors. Three TFCE repeated-measures ANOVAs tested the main effects of Task Sequence and, respectively, CTE, PRE, and RS (Response Sequence) as well as their interaction. Paired-sample *t*-tests were used to analyse ERP correlates of switch costs (switch vs. repeat trials) within each condition that was derived from all six possible combinations of CTE and Response Variability {CTi-PRi | response alternation, CTe-PRi | response alternation, CTi-PRe | response alternation, CTe-PRe | response alternation, (CTi-PRe | response repetition, CTe-PRe | response repetition}.

Cue-locked analyses were performed in the time window [0, 1.150 ms] (i.e. including 50 ms after target onset to cover the complete time window that was used for baseline correction of the target-locked epochs). TFCE repeated-measures ANOVA was used to test the main effects of Task Sequence and PRE as well as their interaction. Due to the disparity in the number of trials between PRi and PRe conditions, PRe data were arbitrarily splitted according to response sequence (i.e., the requirement to alternate or repeat responses, which was indicated by the target stimuli), and the Task Sequence × PRE ANOVA was performed twice, once for each PRe subset (i.e., PRe | response alternation, PRe | response repetition). Results were corrected using Bonferroni correction.

## Results

### Behavioural results

**Figure 4:**
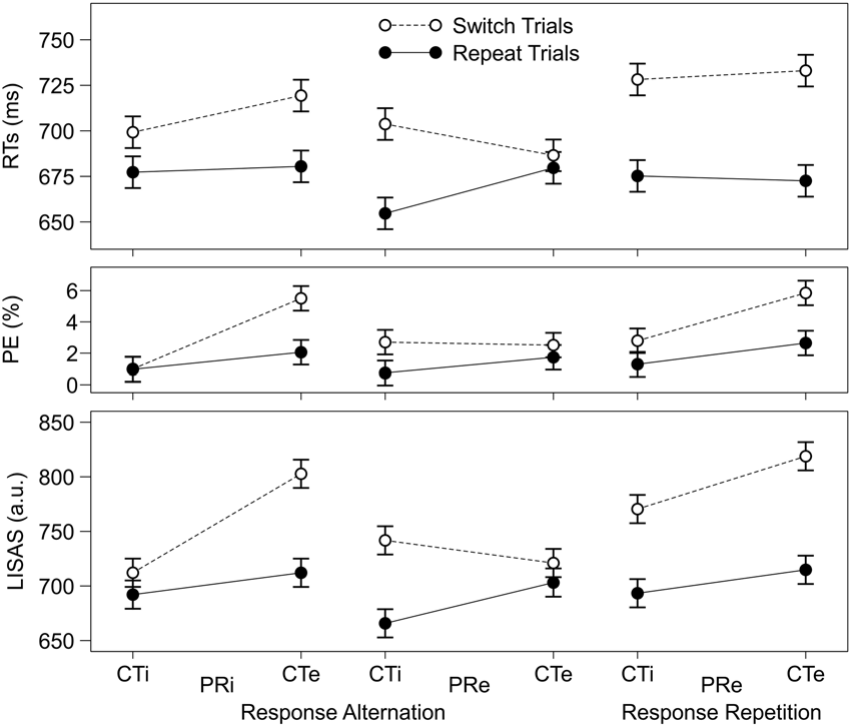
Mean RTs, PE and LISAS (a.u., arbitrary units) separately for task-switch and task-repeat trials, and across CTE (ineligible, eligible) and Response Variability (PRi | response alternation, PRe | response alternation, PRe | response repetition). Error bars represent the 95 percent repeated-measures confidence interval^57^ for the omnibus three-way interaction effect of Task Sequence, CTE, and Response Variability. CTE = competitor-task eligibility; CTe = competitor task eligible; CTi = competitor task ineligible; PRe = previous response eligible; PRi = previous response ineligible; RTs = response times; PE = percent errors; LISAS = linear integrated speed-accuracy score.

Figure 4 displays behavioural data for the three dependent variables (RTs, PE, LISAS) separately for Task Sequence (repeat, switch), CTE (ineligible, eligible), and the nested factor Response Variability (PRi | response alternation, PRe | response alternation, PRe | response repetition) (cf. Supplementary Table S6 for the numerical data). Latency, accuracy, and integrated switch costs are easily graspable by column-wise comparisons between the black entries and the white entries.

### Omnibus ANOVA

We commenced statistical inference with three three-way repeated-measures omnibus ANOVAs (Table 1). Task Sequence (repeat, switch), CTE (ineligible, eligible), and Response Variability (PRi | response alternation, PRe | response alternation, PRe | response repetition) served as repeated-measures variables. RTs, PE, and LISAS served as dependent variables. The mean number of correct trials per cell after exclusion of all to-be-excluded trials (see Methods for criteria) was 81.9–86.3 (minimum number of trials per cell 57–78).

**Table 1:** Omnibus ANOVA results for Task Sequence (repeat, switch), CTE (ineligible, eligible), and Response Variability (PRi | response alternation, PRe | response alternation, PRe | response repetition) for RTs, PE, and LISAS.

	*df1*	*df2*	RTs			PE			LISAS		
			*F*	*p*	η_*p*_^2^	*F*	*p*	η_*p*_^2^	*F*	*p*	η_*p*_^2^
TS	1	35	83.12	<0.001	0.70	25.58	<0.001	0.42	85.15	<0.001	0.71
CTE	1	35	4.03	0.053	0.10	37.13	<0.001	0.52	66.20	<0.001	0.65
RV	2	34	9.91	0.001	0.22	12.90	<0.001	0.27	19.99	<0.001	0.36
TS × CTE	1	35	2.35	0.135	0.06	11.85	0.002	0.25	3.24	0.081	0.09
TS × RV	2	34	7.75	0.001	0.18	3.20	0.049	0.08	10.82	<0.001	0.24
CTE × RV	2	34	1.50	0.231	0.04	18.76	<0.001	0.35	18.94	<0.001	0.35
TS × CTE × RV	2	34	14.00	<0.001	0.29	12.06	<0.001	0.26	27.23	<0.001	0.44

*Note*. TS = Task Sequence; CTE = competitor-task eligibility; RV = Response Variability; RTs = response times; PE = percent errors; LISAS = linear integrated speed-accuracy score.

For all dependent variables, the main effect of Task Sequence proved statistically significant, with overall faster and more accurate responses on repeat trials (latency switch costs: *M* = 38 ms, *SE* = 4; accuracy switch costs: *M* = 1.82%, *SE* = 0.36; integrated switch costs: *M* = 64 a.u., *SE* = 7; Fig. 4). Of more importance were the statistically significant interactions involving Task Sequence that were obtained, with the highest-order interaction being Task Sequence × CTE × Response Variability. We did not parse this three-way interaction into its constituents because the nested factor, Response Variability, complicated these analyses. For that reason, follow-up analyses were fragmented as follows.

### Stimulus-related behavioural switch costs

The Task Sequence by CTE interaction, when exclusively considered on PRi trials, is informative with regard to the occurrence of stimulus-related switch costs (ineligibility of previous responses should eliminate response-related switch costs). We had observed in our previous study^14^ that behavioural switch costs on those trials were strongly modulated by CTE, such that no switch costs were discernible as long as the competitor task remained ineligible, whereas prominent switch costs emerged when the competitor task was eligible.

Inspection of Table 2 reveals that the previous behavioural findings^14^ (*N* = 95) on PRi trials were replicated in the present study (*N* = 36). For latency switch costs, we observed a statistically significant interaction Task Sequence × CTE, indicating modulatory effects of competitor task eligibility on latency switch costs (CTi: *M* = 22 ms, *SE* = 5; CTe: *M* = 39 ms, *SE* = 8; see Fig. 4). For accuracy switch costs, we observed a statistically significant two-way interaction Task Sequence × CTE, indicating modulatory effects of competitor task eligibility on accuracy switch costs (CTi: *M* = 0.02%, *SE* = 0.28; CTe: *M* = 3.45%, *SE* = 0.66; see Fig. 4). For integrated switch costs, we observed a statistically significant two-way interaction, indicating modulatory effects of competitor task eligibility on integrated switch costs (CTi: *M* = 20 a.u., *SE* = 7; CTe: *M* = 91 a.u., *SE* = 14; see Fig. 4). Taken together, the present findings replicate previous ones^14^. They indicate that behavioural switch costs are reliably stronger when competitor tasks are eligible compared to when they remain ineligible. Note that only PRi trials were considered here in order to isolate stimulus-related switch costs.

**Table 2:** ANOVA results for Task Sequence (repeat, switch) and CTE (ineligible, eligible) for RTs, PE, and LISAS on previous response ineligible (PRi) trials.

	*df1*	*df2*	RTs			PE			LISAS		
			*F*	*p*	η_*p*_^2^	*F*	*p*	η_*p*_^2^	*F*	*p*	η_*p*_^2^
TS	1	35	30.92	<0.001	0.47	21.13	<0.001	0.38	40.44	<0.001	0.54
CTE	1	35	5.90	0.020	0.14	53.67	<0.001	0.61	78.07	<0.001	0.69
TS × CTE	1	35	5.49	0.025	0.14	25.50	<0.001	0.42	28.69	<0.001	0.45

*Note*. TS = Task Sequence; CTE = competitor-task eligibility; RTs = response times; PE = percent errors; LISAS = linear integrated speed-accuracy score.

### Response-related behavioural switch costs

The easiest way to accomplish an analysis of potential effects exerted by response-task bindings from preceding trials on behavioural switch costs is by introducing eligibility of the previously executed response. Inspection of Table 3 reveals that switch costs were unaffected by the PRE manipulation (all *F* < 1.08), indicating that manipulating the eligibility of the previous response alone (i.e., holding constant response alternations) has no effect on latency switch costs (PRi: *M* = 30 ms, *SE* = 5; PRe: *M* = 28 ms, *SE* = 6), accuracy switch costs (PRi: *M* = 1.73%, *SE* = 0.38; PRe: *M* = 1.37%, *SE* = 0.41), and integrated switch costs (PRi: *M* = 55 a.u., *SE* = 9; PRe: *M* = 47 a.u., *SE* = 9). In contrast, inspection of Table 4 reveals that switch costs were affected by the response sequence manipulation (all *F* > 5.67), indicating that response repetitions were associated with more pronounced latency switch costs (*M* = 57 ms, *SE* = 7), accuracy switch costs (*M* = 2.35%, *SE* = 0.49), and integrated switch costs (*M* = 91 a.u., *SE* = 10) compared to response alternations (latency switch costs: *M* = 28 ms, *SE* = 6; accuracy switch costs: *M* = 1.37%, *SE* = 0.41; integrated switch costs: *M* = 47 a.u., *SE* = 9; see also Fig. 4, Supplementary Table S6). These results do not only replicate our previous findings^14^, but they are also in good agreement with the task-switching literature, as described in the Introduction. They indicate that behavioural switch costs are reliably stronger when responses need to be repeated compared to when responses alternate across trials (when eligibility of previous responses is kept constant).

**Table 3:** ANOVA results for Task Sequence (repeat, switch), CTE (ineligible, eligible), and PRE (ineligible, eligible) for RTs, PE, and LISAS on response-alternation trials.

	*df1*	*df2*	RTs			PE			LISAS		
			*F*	*p*	η_*p*_^2^	*F*	*p*	η_*p*_^2^	*F*	*p*	η_*p*_^2^
TS	1	35	46.24	<0.001	0.57	19.71	<0.001	0.36	49.70	<0.001	0.59
CTE	1	35	5.82	0.021	0.14	30.70	<0.001	0.47	56.58	<0.001	0.62
PRE	1	35	10.76	0.002	0.24	5.12	0.030	0.13	13.87	0.001	0.28
TS × CTE	1	35	5.18	0.029	0.13	5.33	0.027	0.13	0.50	0.483	0.01
TS × PRE	1	35	0.09	0.763	<0.01	1.07	0.309	0.03	0.77	0.388	0.02
CTE × PRE	1	35	1.39	0.246	0.04	61.96	<0.001	0.64	31.37	<0.001	0.47
TS × CTE × PRE	1	35	28.02	<0.001	0.45	19.48	<0.001	0.36	44.45	<0.001	0.56

*Note*. TS = Task Sequence; CTE = competitor-task eligibility; PRE = previous response eligibility; RTs = response times; PE = percent errors; LISAS = linear integrated speed-accuracy score.

**Table 4:** ANOVA results for Task Sequence (repeat, switch), CTE (ineligible, eligible), and Response Sequence (alternation, repetition) for RTs, PE, and LISAS on previous response eligible trials.

	*df1*	*df2*	RTs			PE			LISAS		
			*F*	*p*	η_*p*_^2^	*F*	*p*	η_*p*_^2^	*F*	*p*	η_*p*_^2^
TS	1	35	65.58	<0.001	0.65	22.00	<0.001	0.39	83.41	<0.001	0.70
CTE	1	35	0.48	0.492	0.01	19.35	<0.001	0.36	18.97	<0.001	0.35
RS	1	35	13.01	0.001	0.27	20.43	<0.001	0.37	27.27	<0.001	0.44
TS × CTE	1	35	10.42	0.003	0.23	0.51	0.479	0.01	3.21	0.082	0.08
TS × RS	1	35	12.12	0.001	0.26	5.67	0.023	0.14	17.15	<0.001	0.33
CTE × RS	1	35	0.29	0.596	0.01	15.19	<0.001	0.30	18.45	<0.001	0.35
TS × CTE × RS	1	35	15.63	<0.001	0.31	6.82	0.013	0.16	24.13	<0.001	0.41

*Note*. TS = Task Sequence; RS = Response Sequence; CTE = competitor-task eligibility; RTs = response times; PE = percent errors; LISAS = linear integrated speed-accuracy score

### Interactions between stimulus- and response-related switch costs

Another issue is whether there is evidence for interactions between stimulus- and response-related switch costs. The easiest way to accomplish an analysis of potential interaction effects on behavioural switch costs is by introducing both manipulations, namely a) competitor task eligibility and b) eligibility of the previously executed response. Inspection of Table 3 reveals that the switch costs were affected by the CTE by PRE interaction (all *F* > 19.47). Inspection of Fig. 4 and Supplementary Table S6 reveals that competitor task eligibility was associated with more pronounced switch costs on PRi trials, as discussed above. However, this fact stands vis-à-vis to the absence of switch cost enhancements with competitor task eligibility on PRe trials (latency switch costs CTi: *M* = 49 ms, *SE* = 9; CTe: *M* = 7 ms, *SE* = 6; accuracy switch costs CTi: *M* = 1.96%, *SE* = 0.63; CTe: *M* = 0.78%, *SE* = 0.46; integrated switch costs CTi: *M* = 76 a.u., *SE* = 12; CTe: *M* = 18 a.u., *SE* = 10). The presence of this three-way interaction (Task Sequence, CTE, PRE) indicates that the modulatory influence of CTE on behavioural switch costs, that was clearly discernible on PRi trials, is eliminated (or even reversed) on PRe trials.

### ERP Results

#### Target-locked ERPs

**Figure 5:**
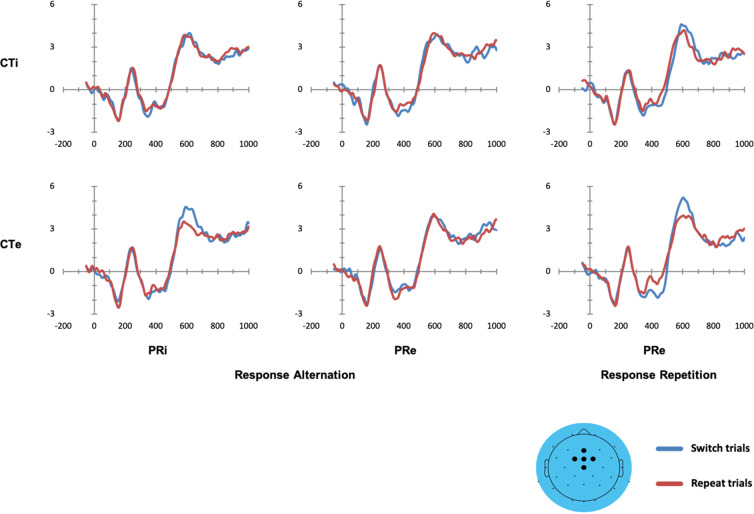
Target-locked reconstructed grand-average ERP waveforms, separately for all 12 experimental conditions at fronto-central electrodes as indicated in the scalp map (black dots). Upper panels: CTi, competitor task ineligible. Lower panels: CTe, competitor task eligible. Left-sided panels: PRi, previous response ineligible. Central panels: PRe, previous response eligible, response alternation. Right-sided panels: PRe, previous response eligible, response repetition. Task switch (blue lines) and task repeat (red lines) trials as indicated.

Figure 5 shows the target-locked reconstructed ERP waveforms after application of RIDE-based latency-jitter correction of R- and C-cluster waveforms, separately for all 12 experimental conditions (see Supplementary Fig. S1 for comparison with conventionally averaged ERP waveforms). Inspection of Fig. 5 reveals that no switch-related ERP modulations seemed to emerge on any CTi condition. In contrast, there seemed to emerge two distinct switch-related ERP modulations on CTe conditions: First, a switch-related enhancement of P3 amplitudes at fronto-central electrodes became visible. The switch-related P3 enhancement seemed to be present when previous responses remained ineligible (i.e., on PRi trials) and when responses had to be repeated (i.e., on PRe | response repetition trials), while no signs of switch-related P3 enhancement appeared on PRe | response alternation trials. Second, a switch-related enhancement of N2 amplitudes at fronto-central electrodes was visible when previous responses had to be repeated on switch trials (i.e., on PRe | response repetition trials).

**Figure 6:**
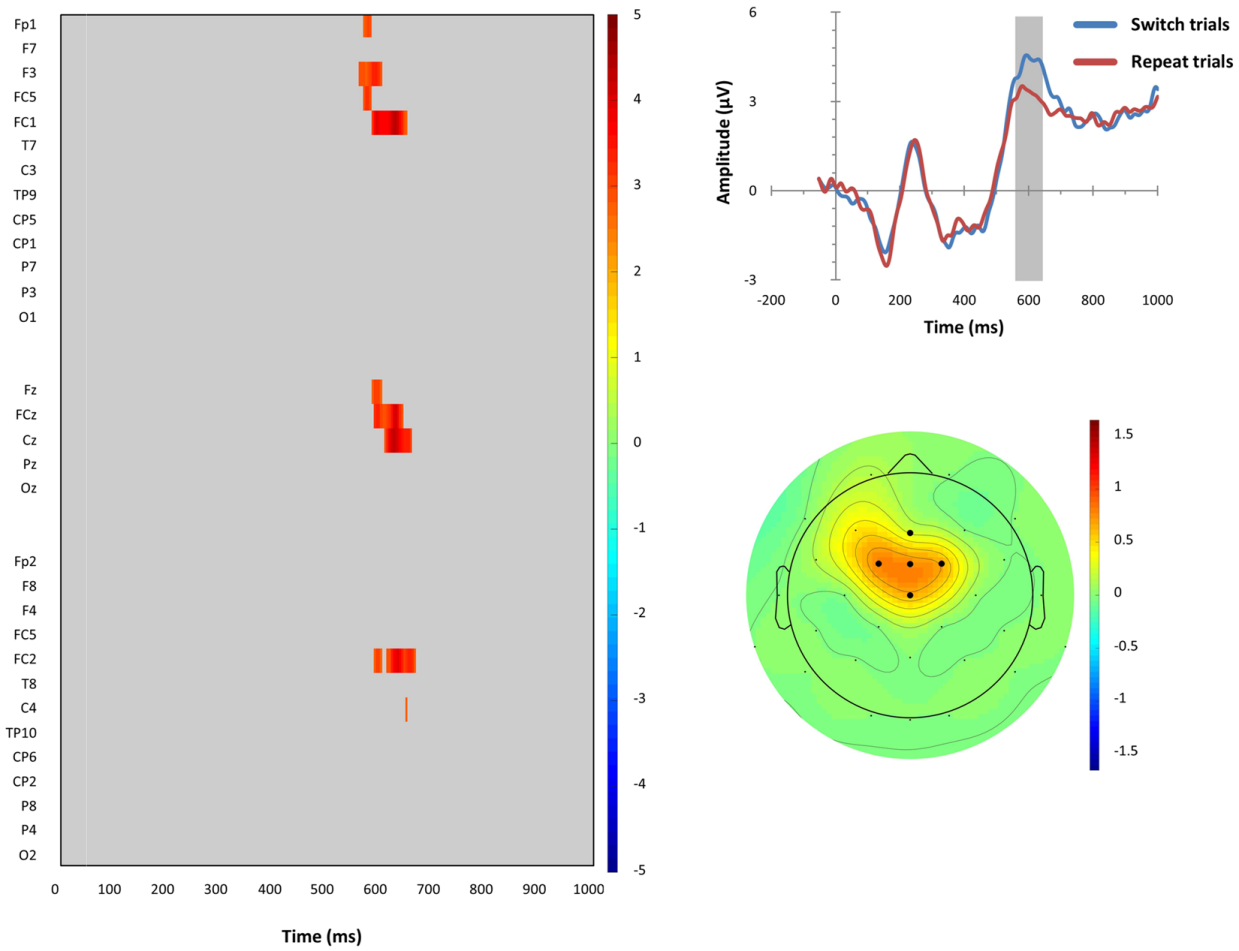
Left-sided panel: Raster diagram showing time course and topographic distribution of significant effects in target-locked reconstructed grand-average ERP waveforms that were elicited by task switches in comparison to task repeats (color-coded *t* values). Only trials on which competitor tasks remained eligible (CTe), but previous responses were ineligible (PRi), are considered here. Right-sided panels: Upper panel: Target-locked reconstructed grand-average ERP waveforms, separately for task switch and task repeat trials at the fronto-central electrodes as indicated in the scalp map below (black dots). Lower panel: The scalp topography of the switch-related P3-like amplitude differences (in μV).

The three repeated-measures ANOVAs, Task Sequence × CTE, Task Sequence × PRE, and Task Sequence × RS, revealed the presence of statistically significant switch-related modulation of reconstructed ERP waveforms (results are reported in Supplementary Figs. S2 and S3, respectively), but no other significant effect. Pairwise comparisons confirmed significant switch-related ERP waveform modulations in only two specific contrasts: First, in the CTe condition when the previous response remained ineligible (PRi), switch trials compared to repeat trials showed a statistically reliable enhanced P3-like positive deflection at fronto-central electrodes (F3, FC1, FCz, FC2, Cz) at around 560–660 ms post-stimulus (see Fig. 6). These statistical findings corroborated the educated guess that could be obtained from visual inspection of reconstructed P3 waveforms on CTe | PRi trials that are visible in the lower left panel of Fig. 5. In contrast, statistical findings did not corroborate the educated guess that could be obtained from visual inspection of the reconstructed P3 waveforms on CTe | PRe | response repetition trials that are visible in the lower right panel of Fig. 5.

**Figure 7:**
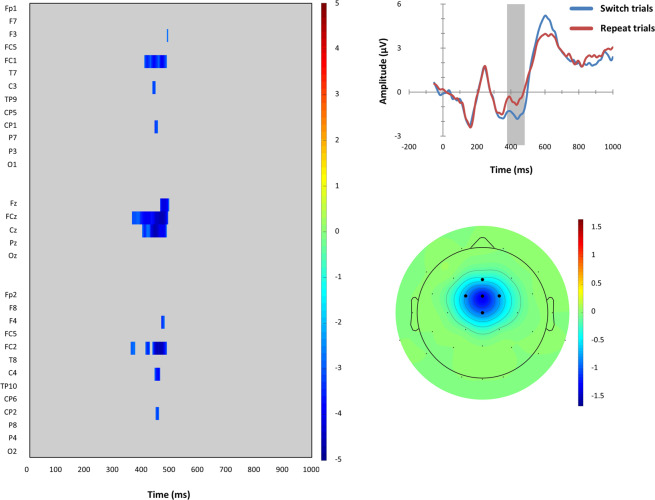
Left-sided panel: Raster diagram showing time course and topographic distribution of significant effects in target-locked reconstructed grand-average ERP waveforms that were elicited by task switches in comparison to task repeats (color-coded *t* values). Only trials on which competitor tasks remained eligible (CTe), previous responses were eligible (PRe), and responses had to be repeated are considered here. Right-sided panels: Upper panel: Target-locked reconstructed grand-average ERP waveforms, separately for task switch and task repeat trials at the fronto-central electrodes as indicated in the scalp map below (black dots). Lower panel: The scalp topography of the switch-related N2-like amplitude differences (in μV).

Second, on CTe | PRe | response repetition trials, switch trials showed an enhanced N2-like negative deflection compared to repeat trials at fronto-central electrodes (Fz, FC1, FCz, FC2, Cz) at around 370–490 ms (see Fig. 7). These statistical findings corroborated the educated guess that could be obtained from mere visual inspection of the reconstructed ERPs shown in the lower right panel of Fig. 5.

#### Cue-locked ERPs

**Figure 8:**
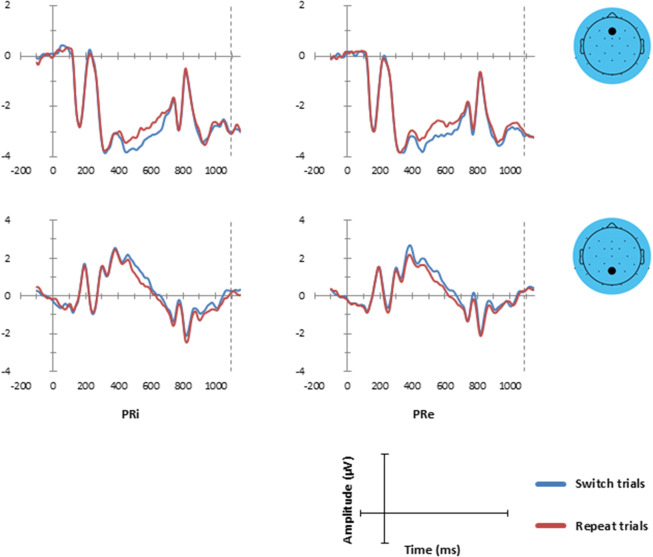
Cue-locked reconstructed grand-average ERP waveforms, separately for the four cue-locked suitable experimental conditions at electrodes as indicated in the scalp maps (black dots). Left-sided panels: PRi, previous response ineligible. Right-sided panels: PRe, previous response eligible. Task switch (blue lines) and task repeat (red lines) trials as indicated.

Figure 8 shows the cue-locked reconstructed ERP waveforms after the application of RIDE-based latency-jitter correction of one C-cluster waveform, separately for those four experimental conditions for which cue-locking was meaningful (see Supplementary Fig. S4 for comparison with conventionally averaged ERP waveforms). Cue-locking is exclusively meaningful in those four conditions because CTE and response sequence remain unknowledgeable at cue onset. Inspection of Fig. 8 suggested the presence of ERP waveform alterations related to switch cues in both PRE conditions.

**Figure 9:**
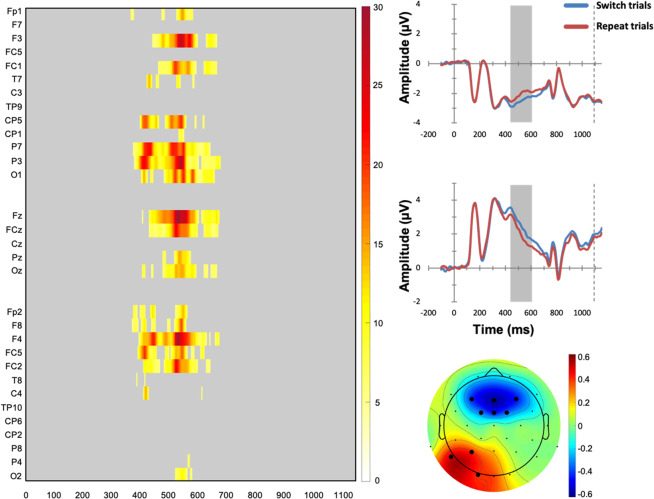
Left-sided panel: Raster diagram showing time course and topographic distribution of significant main effect for Task Sequence in cue-locked reconstructed grand-average ERP waveforms (color-coded *F* values, alpha-corrected = 0.025) for one of the two conducted ANOVAs (see Supplementary Fig. S5, for the results of the other ANOVA). Right-sided panels: Upper panel: Cue-locked reconstructed grand-average ERP waveforms, separately for task switch and task repeat trials at frontal and posterior electrodes as indicated in the scalp map below (black dots). Lower panel: The scalp topography of the switch-related amplitude differences (in μV).

The Task Sequence × PRE ANOVAs revealed the presence of statistically significant switch-related ERP modulations on cue-locked reconstructed ERP waveforms (see Fig. 9 and Supplementary Fig. S5). Switch trials elicited a more negative reconstructed ERP waveform at fronto-central electrodes (Fp1, Fp2, F3, Fz, F4, F8, FC1, FCz, FC2, FC5), and also a more positive reconstructed ERP waveform at parietal electrodes (CP5, CP2, P7, P3, Pz, Oz, O2), around 360–650 ms after cue onset (see Fig. 9).

## Discussion

The present data are congruent with the DH, which traces behavioural switch costs back to stimulus-related and response-related cognitive interference^11^. According to the DH, these two sources of cognitive interference should be associated with (at least partially) separable neural networks. As hypothesized, two distinct ERP signatures of stimulus-related and response-related switch costs arose: Purely stimulus-related switch costs occurred when competitor tasks remained eligible while previous responses were ineligible, and enhanced P3-like ERP amplitudes co-occurred with their emergence. Response-related switch costs occurred when previously executed responses had to be repeated on switch trials, and enhanced N2-like ERP amplitudes co-occurred with their emergence. These two ERP waveform modulations, however, were best observable following RIDE-based latency-jitter correction^38^ of the original trial-by-trial ERP waveforms. As suggested by the authors, RIDE-based latency-jitter correction therefore represents a convenient methodological refinement for ERP analyses^38,58–60^. RIDE-based latency-jitter correction seems to be important in task-switching studies (see also^61,62^), probably due to the typically relatively slow and variable trial-by-trial RTs in task-switching studies. We discuss behavioural, target-locked and cue-locked ERP data separately in the following paragraphs.

The behavioural data (see Fig. 4) replicate our previously reported results^14^, and they lend support to the DH. Manipulating competitor-task eligibility exerted strong effects on behavioural switch costs when previous responses remained ineligible, thereby diminishing potential response-related switch costs (i.e., on PRi trials). Weak switch costs were observed when competitor tasks were ineligible (i.e., on CTi trials), but switch costs were substantial when competitor tasks remained eligible (i.e., on CTe trials). This data pattern indicates massive effects from stimulus-related switch costs. Figure 1 outlines our explanation for this data pattern: It focuses on the reenactment of the previously relevant stimulus-set binding that occurs on CTe (but not on CTi) switch trials, and that exerts proactive interference on those switch trials.

Manipulating competitor-task eligibility also successfully emulated typical effects of stimulus valence/congruency on behavioural switch costs: Targets on CTi trials resembled univalent stimuli, while targets on CTe trials comprised bivalent stimuli. Bivalent targets may be congruent (i.e., both stimulus features are mapped to the same response) or incongruent (i.e., both stimulus features are mapped to different responses). CTe targets resembled bivalent incongruent stimuli because the two features of our targets on CTe trials were always mapped to two different responses in our study. Taken together, these behavioural findings indicate purely stimulus-related switch costs, with massively reduced switch costs following quasi-univalent targets on CTi conditions, while substantial switch costs emerged for bivalent incongruent targets on CTe conditions.

Switch costs were also pronounced on those trials that demanded response repetitions (i.e., on response-repetition switch trials), irrespective of stimulus valence/congruency. This data pattern indicates the effects of response-related switch costs. Figure 1 outlines our explanation for this data pattern: It focuses on the reenactment of the previously relevant response-set binding that occurs on response repetition (but less so on response alternation) switch trials, thereby exerting proactive interference on those switch trials.

The DH^11^ predicted two target-locked ERP correlates of behavioural switch costs. First, stimulus-related switch costs should be correlated with enhanced switch-related P3 amplitudes. More precisely, when previous responses remain ineligible (i.e., on PRi trials), the DH predicted enhanced P3 amplitudes in response to (bivalent incongruent) targets (i.e., on CTe switch trials) compared to (quasi-univalent) targets (i.e., on CTi switch trials). Second, response-related switch costs should be correlated with enhanced N2 amplitudes. That is, enhanced N2 amplitudes were expected on those switch trials on which previously executed responses had to be repeated.

Enhanced target-locked P3-like amplitudes were observed on switch trials compared to repeat trials in that CTe condition which minimized response-related switch costs (i.e., in PRi conditions; see Fig. 6). These switch-related effects on P3-like amplitudes parallel the robust behavioural switch costs that occur in the CTe-PRi condition. Thus, behavioural switch costs and electrophysiological switch-related ERP modulations follow our manipulation of competitor-task eligibility, and they occur preferentially when contributions to switch costs from response-related sources are at minimum (i.e., they occur preferentially in PRi conditions). This data pattern is clearly congruent with the DH by indicating that stimulus-related switch costs are associated with variations in P3-like amplitudes.

Enhanced target-locked N2-like amplitudes were observed on switch trials compared to repeat trials in the CTe condition when response repetitions were required (see Fig. 7). Thus, we found switch-related effects on N2-like amplitudes when competitor-tasks remained eligible and responses had to be repeated. This data pattern, in which the coincidence of stimulus- and response-related switch costs seems to be associated with variations in N2-like amplitudes, is partly congruent with the DH. One way to interpret the N2 amplitude enhancement in the present study is that it is associated with the resolution of response-related cognitive interference^63–66^.

Many previous ERP studies of task switching were primarily analysing cue-locked ERPs, possibly because they provide indices of neural correlates of proactive task preparation (for overview see^67^). Sustained switch-related modulations of cue-locked ERPs provided evidence that switch cues received neural processing that differed in some way from that of repeat cues, as described in detail in Figs. 8, 9. Previous research repeatedly showed similar sustained switch-related cue-locked ERP modulations, in particular the occurrence of frontal negative and of parietal positive ERP waveforms^68,69^. The main issue related to these switch-cue ERP modulations is whether or not they are exclusively reflecting proactive task preparation. It has been suggested that switch-cue ERP modulations may also reflect sequence effects on switch-cue processing to some degree. In its simplest form, switch-cue sequences may be considered as ‘oddball’ series of events, with repeat cues resembling standard oddballs, and switch cues representing target ‘oddballs’ (e.g.,^70^).

Response eligibility did not seem to exert detectable effects on cue-locked ERPs. Albeit it is difficult interpreting negative ERP findings, the absence of response eligibility effects on cue-locked ERP waveforms does not seem to provide electrophysiological evidence for more effective task preparation when previously executed responses remained eligible on the upcoming trial. Thus, response-related switch costs may reflect reactive reconfiguration of task sets following target^32^.

We reached the reported results by manipulating competitor-task eligibility and response eligibility in a card-matching task via spatially arranged response cues. Among the open issues is whether the present findings, provided their replicability, would generalize to other, superficially dissimilar task-switching paradigms. It remains to be shown whether other experimental manipulations of stimulus-related and response-related switch costs will exert similar effects on ERPs.

Another issue concerns potential theoretical implications of the present findings. Other nomenclatures may be more or less closely related to our notion of stimulus-related and response-related switch costs, such as shifting attentional and intentional set^71,72^. These terms may refer to a common denominator, which may be the distinction between switch costs arising from afferent (i.e., neural networks that are rooted in from-receptors-to-brain pathways) or efferent (i.e., neural networks that are rooted in from-brain-to-effectors pathways) circuitry in the brain.

Related to nomenclature, we wish to clarify that the term ‘stimulus-related’ should be treated with caution. The term ‘stimulus’ refers here to stimulus evaluation plus response selection, and it may be best considered as comprising *premotor* aspects of information processing. This clarification is due to the fact that stimulus eligibility refers to a congruency effect (i.e., bivalent incongruent vs. univalent) rather than a valence effect (i.e., bivalent congruent vs. univalent) in our study. Hence, the manipulation of stimulus eligibility clearly incorporates aspects of response selection in addition to stimulus evaluation in our study. In contrast, the term ‘response-related’ is associated with our manipulation of response eligibility, in particular with the sequential manipulation of response repetition/alternation. Therefore, it may be best considered as comprising *motor* aspects of information processing.

These considerations further qualify our reasoning that was outlined in Fig. 1. The reenactment of the previously relevant stimulus-set binding should not only include stimulus features, but also response properties at the conceptual, premotor level. In contrast, the reenactment of the previously relevant response-set binding should only include response properties at the motor level.

There are also methodological concerns, most notably that the full ERP data pattern was best observable following RIDE-based latency-jitter correction of the original ERP waveforms^38^. As already noted, RIDE-based latency-jitter correction therefore represents an important methodological refinement of ERP analyses of cognitive task switching (see also^61,62^). This seems to be due to the fact that complex cognitive processes, such as switching between cognitive tasks, do almost never occur in temporal synchrony with stimulus onset. Rather than that, they occur with considerable trial-by-trial latency variability, rendering latency-jitter correction an indispensable necessity.

And yet, RIDE-based latency-jitter correction leaves potential overlap between ERP components uncorrected. For example, switch-related effects on CTe/RR trials were visible in the N2 and in the P3 latency range (in fact, the confluence of both waveform modulations was predicted be the DH), but only the switch-related N2 modulation reached statistical significance in these analyses. It is however easily conceivable that the occurrence of a negative and positive waveform modulation in close spatio-temporal proximity might have given rise to component overlap, such that this overlap primarily affected the P3 waveform modulation (through overlay of a more sustained negative waveform deflection). We have to leave open the methodological issue how latency-jitter correction can be combined with the temporal identification of ERP components in future studies.

These data indicate that the DH^11^ possesses some credibility. The behavioural switch costs supported the presence of dissociable stimulus-related and response-related switch costs^14^. The cue-locked ERP data suggest enhanced neural recruitment for task preparation in response to switch cues. The proactive cue-locked ERP switch effects occurred irrespective of response eligibility, but further research is needed to confirm this conclusion. The target-locked ERP data clearly support the distinction between stimulus- and response-related switch costs, and they substantiate the claim that their neural underpinnings are at least partially separable. We found an ERP correlate of purely stimulus-related task-set interference (i.e., P3 waveforms with a fronto-central scalp distribution). A strikingly different ERP correlate emerged when stimulus-related and response-related sources of task-set interference coincided (i.e., N2 waveforms with a fronto-central scalp distribution). Our conclusion from the present target-locked ERP data is that - at least partially - separable neural networks are involved in resolving stimulus-related and response-related switch costs.

## Supplementary information

Supplementary information.

## Data Availability

The datasets analysed during the current study are available from the corresponding author on reasonable request.
