# Supplementary material for: Cognitive flexibility and N2/P3 event-related brain potentials
Source: Sci Rep. 2020 Jun 17;10:9859. doi: 10.1038/s41598-020-66781-5 (PMC7299939; doi:10.1038/s41598-020-66781-5)
Supplement: Supplementary file 1 — Supplementary information. [file 41598_2020_66781_MOESM1_ESM.docx]

**Cognitive flexibility and N2/P3 event-related brain potentials**

**- Supplementary Materials -**

Bruno Kopp*, Alexander Steinke, Antonino Visalli

Department of Neurology, Hannover Medical School; Carl-Neuberg-Straße 1, 30625 Hannover, Germany

*Correspondence concerning this article should be addressed to Bruno Kopp, Department of Neurology, Hannover Medical School, Carl-Neuberg-Str. 1, 30625 Hannover, Germany. Tel. +49-511-5322439. E-mail: [kopp.bruno@mh-hannover.de](mailto:kopp.bruno@mh-hannover.de)

Table S1

*Number of epochs that were excluded from data analysis.*

|  | Target | | | | Cue | | |
| --- | --- | --- | --- | --- | --- | --- | --- |
| ID | Timeout | Error | Bad Trials | Union | Error | Bad Trials | Union |
| 2 | 8 | 11 | 62 | 77 | 11 | 69 | 86 |
| 3 | 9 | 47 | 64 | 139 | 47 | 64 | 145 |
| 4 | 13 | 36 | 59 | 126 | 36 | 52 | 120 |
| 5 | 4 | 23 | 50 | 91 | 23 | 41 | 84 |
| 6 | 3 | 54 | 71 | 163 | 54 | 71 | 164 |
| 7 | 4 | 10 | 62 | 80 | 10 | 65 | 82 |
| 9 | 1 | 18 | 56 | 78 | 18 | 58 | 89 |
| 10 | 4 | 7 | 53 | 61 | 7 | 49 | 58 |
| 11 | 14 | 9 | 30 | 58 | 9 | 33 | 51 |
| 12 | 1 | 15 | 53 | 81 | 15 | 55 | 76 |
| 13 | 24 | 63 | 81 | 212 | 63 | 76 | 193 |
| 14 | 3 | 13 | 37 | 62 | 13 | 40 | 64 |
| 15 | 1 | 17 | 66 | 75 | 17 | 52 | 80 |
| 16 | 5 | 25 | 51 | 95 | 25 | 57 | 99 |
| 17 | 2 | 33 | 78 | 137 | 33 | 63 | 120 |
| 18 | 0 | 3 | 67 | 68 | 3 | 97 | 101 |
| 19 | 0 | 28 | 56 | 95 | 28 | 53 | 105 |
| 20 | 13 | 61 | 37 | 154 | 61 | 49 | 164 |
| 21 | 15 | 36 | 244 | 308 | 36 | 271 | 313 |
| 23 | 26 | 14 | 48 | 95 | 14 | 55 | 78 |
| 25 | 0 | 32 | 118 | 147 | 32 | 125 | 174 |
| 26 | 6 | 39 | 53 | 114 | 39 | 53 | 124 |
| 27 | 0 | 47 | 50 | 134 | 47 | 46 | 130 |
| 28 | 12 | 5 | 33 | 44 | 5 | 42 | 51 |
| 29 | 1 | 4 | 89 | 92 | 4 | 133 | 129 |
| 30 | 0 | 6 | 50 | 54 | 6 | 46 | 53 |
| 31 | 3 | 22 | 65 | 92 | 22 | 57 | 94 |
| 32 | 1 | 34 | 52 | 112 | 34 | 63 | 120 |
| 33 | 2 | 4 | 172 | 175 | 4 | 192 | 184 |
| 34 | 0 | 58 | 103 | 166 | 58 | 97 | 186 |
| 35 | 20 | 62 | 76 | 200 | 62 | 92 | 204 |
| 36 | 15 | 3 | 25 | 44 | 3 | 32 | 36 |
| 37 | 8 | 27 | 78 | 126 | 27 | 67 | 116 |
| 38 | 1 | 120 | 34 | 257 | 120 | 29 | 251 |
| 39 | 0 | 21 | 57 | 97 | 21 | 47 | 86 |
| 40 | 2 | 33 | 61 | 118 | 33 | 50 | 112 |
| *Mean* | 6.14 | 28.89 | 67.81 | 117.42 | 28.89 | 70.58 | 120.06 |
| *SD* | 7.14 | 24.09 | 40.44 | 58.92 | 24.09 | 46.77 | 59.52 |
| *Min* | 0 | 3 | 25 | 44 | 3 | 29 | 36 |
| *Max* | 26 | 120 | 244 | 308 | 120 | 271 | 313 |

*Note*. Timeout = Trials on which the RT was slower than 2 sec (only for the target-locked analysis); Error = error + post-error (errors and post-errors led to exclusion of target- and cue-locked epochs on these trials); Bad trials = automatically-identified and manually identified EEG outlier trials. "Union" represents the union (i.e., the same trial could have been marked as error and bad trial, but it was counted just once in the aggregated number of rejected epochs); *SD* = standard deviation.

Table S2

*Condition-wise Pearson correlations between RTs and PE.*

| Task Sequence | CTE | Response Sequence | PRE | *r* | *p* |
| --- | --- | --- | --- | --- | --- |
| Repeat | Ineligible | Alternation | Ineligible | .088 | .611 |
|  |  |  | Eligible | .037 | .829 |
|  |  | Repetition | Eligible | .031 | .856 |
|  | Eligible | Alternation | Ineligible | -.023 | .893 |
|  |  |  | Eligible | .010 | .955 |
|  |  | Repetition | Eligible | .253 | .137 |
| Switch | Ineligible | Alternation | Ineligible | .030 | .860 |
|  |  |  | Eligible | .077 | .656 |
|  |  | Repetition | Eligible | .069 | .688 |
|  | Eligible | Alternation | Ineligible | .035 | .840 |
|  |  |  | Eligible | -.060 | .728 |
|  |  | Repetition | Eligible | -.059 | .734 |

*Note.* Two-sided *p*-values are reported. RTs = response times; PE = percent errors; CTE = competitor-task eligibility; PRE = previous response eligibility.

Table S3

*Results of the linear mixed model conducted on LISAS.*

| Fixed Effects | | | |
| --- | --- | --- | --- |
| Predictors | Estimates | CI | *p* |
| (Intercept) | 723.78 | 675.02 – 772.54 | < .001 |
| TS | 176.71 | 131.56 – 221.85 | < .001 |
| CTE | 634.06 | 588.91 – 679.21 | < .001 |
| PRE | -8.98 | -35.05 – 17.09 | .500 |
| RS | -11.70 | -37.76 – 14.37 | .379 |
| TS * CTE | -774.02 | -837.87 – -710.17 | < .001 |
| TS * PRE | -72.72 | -109.58 – -35.86 | < .001 |
| CTE * PRE | -656.77 | -693.63 – -619.91 | < .001 |
| TS * RS | -86.06 | -122.93 – -49.20 | < .001 |
| CTE * RS | -680.36 | -717.23 – -643.50 | < .001 |
| (TS * CTE) * PRE | 690.05 | 637.92 – 742.19 | < .001 |
| (TS * CTE) * RS | 759.36 | 707.23 – 811.49 | < .001 |
| Random Effects | | | |
| Predictor | Estimates |  |  |
| σ^2^ | 3183.68 |  |  |
| τ_ID_ | 12732.63 |  |  |
| ICC | 0.80 |  |  |
| N_ID_ | 36 |  |  |
| Observations | 432 |  |  |
| Marginal R^2^ / Conditional R^2^ | 0.726 / 0.945 |  |  |

*Note.* The *Fixed Effects* part reports the estimated fixed effects coefficients, including confidence intervals (CI) and p-values. The *Random Effects* part reports the model’s group count (N_ID_, amount of random intercepts) as well as the Intra-Class-Correlation-Coefficient (ICC) and information on the random effect within-group (σ^2^) and between-individuals (τ_ID_) variances. Marginal (including fixed effects only) and conditional (including both fixed and random effects) R^2^ are reported. Abbreviations: TS: Task Sequence; CTE: Competitor Task Eligibility; PRE: Previous Response Eligibility; RS: Response Sequence.Table S4

*Number of analysed epochs per participant and condition for the target-locked EEG data analysis.*

|  | Task Repeat | | | | | | Task Switch | | | | | |
| --- | --- | --- | --- | --- | --- | --- | --- | --- | --- | --- | --- | --- |
|  | CTi | | | CTe | | | CTi | | | CTe | | |
|  | RA | RA | RR | RA | RA | RR | RA | RA | RR | RA | RA | RR |
| ID | PRi | PRe | PRe | PRi | PRe | PRe | PRi | PRe | PRe | PRi | PRe | PRe |
| 2 | 87 | 86 | 85 | 81 | 84 | 86 | 81 | 85 | 84 | 82 | 82 | 80 |
| 3 | 80 | 80 | 82 | 78 | 80 | 76 | 80 | 84 | 74 | 72 | 82 | 73 |
| 4 | 79 | 83 | 79 | 84 | 85 | 79 | 82 | 78 | 74 | 73 | 79 | 79 |
| 5 | 83 | 81 | 86 | 80 | 83 | 85 | 87 | 84 | 78 | 80 | 81 | 81 |
| 6 | 77 | 81 | 81 | 77 | 76 | 77 | 79 | 78 | 75 | 72 | 75 | 69 |
| 7 | 86 | 80 | 83 | 80 | 85 | 84 | 84 | 85 | 85 | 82 | 84 | 82 |
| 9 | 85 | 81 | 85 | 84 | 83 | 86 | 85 | 83 | 80 | 83 | 81 | 86 |
| 10 | 85 | 84 | 88 | 83 | 85 | 86 | 85 | 86 | 87 | 85 | 81 | 84 |
| 11 | 86 | 85 | 87 | 83 | 86 | 84 | 87 | 86 | 87 | 82 | 84 | 85 |
| 12 | 85 | 80 | 85 | 82 | 85 | 83 | 89 | 80 | 84 | 81 | 82 | 83 |
| 13 | 76 | 72 | 72 | 73 | 73 | 71 | 72 | 75 | 79 | 69 | 70 | 66 |
| 14 | 84 | 87 | 82 | 86 | 88 | 88 | 85 | 87 | 78 | 84 | 84 | 85 |
| 15 | 86 | 86 | 86 | 85 | 81 | 82 | 84 | 82 | 85 | 81 | 86 | 81 |
| 16 | 80 | 84 | 88 | 79 | 83 | 80 | 82 | 82 | 86 | 81 | 82 | 78 |
| 17 | 76 | 74 | 83 | 80 | 84 | 78 | 83 | 77 | 81 | 77 | 72 | 78 |
| 18 | 84 | 88 | 83 | 84 | 87 | 84 | 84 | 86 | 85 | 81 | 85 | 81 |
| 19 | 85 | 81 | 87 | 82 | 80 | 88 | 85 | 82 | 81 | 76 | 80 | 78 |
| 20 | 78 | 83 | 68 | 80 | 76 | 79 | 71 | 76 | 76 | 68 | 73 | 66 |
| 21 | 62 | 56 | 66 | 68 | 65 | 66 | 67 | 67 | 64 | 61 | 67 | 63 |
| 23 | 83 | 89 | 79 | 85 | 84 | 79 | 82 | 80 | 81 | 79 | 83 | 81 |
| 25 | 82 | 85 | 80 | 75 | 77 | 75 | 79 | 79 | 73 | 77 | 79 | 72 |
| 26 | 85 | 84 | 76 | 85 | 85 | 76 | 80 | 83 | 73 | 77 | 85 | 77 |
| 27 | 79 | 79 | 79 | 80 | 86 | 76 | 82 | 78 | 84 | 68 | 78 | 77 |
| 28 | 86 | 86 | 89 | 86 | 86 | 88 | 83 | 87 | 86 | 86 | 87 | 86 |
| 29 | 77 | 82 | 87 | 85 | 83 | 88 | 83 | 78 | 85 | 80 | 77 | 83 |
| 30 | 88 | 85 | 87 | 85 | 85 | 81 | 86 | 87 | 84 | 86 | 85 | 87 |
| 31 | 84 | 82 | 82 | 83 | 86 | 84 | 85 | 80 | 76 | 81 | 84 | 81 |
| 32 | 82 | 83 | 77 | 80 | 83 | 80 | 78 | 83 | 84 | 80 | 81 | 77 |
| 33 | 73 | 70 | 77 | 79 | 75 | 79 | 77 | 73 | 74 | 75 | 74 | 79 |
| 34 | 75 | 75 | 76 | 76 | 77 | 75 | 82 | 75 | 80 | 73 | 77 | 73 |
| 35 | 78 | 76 | 73 | 67 | 76 | 75 | 80 | 77 | 72 | 72 | 69 | 65 |
| 36 | 83 | 85 | 89 | 86 | 86 | 87 | 87 | 87 | 86 | 88 | 87 | 85 |
| 37 | 82 | 85 | 82 | 78 | 73 | 83 | 81 | 81 | 79 | 78 | 78 | 74 |
| 38 | 74 | 73 | 78 | 71 | 69 | 72 | 74 | 62 | 71 | 63 | 60 | 56 |
| 39 | 81 | 83 | 84 | 81 | 83 | 87 | 79 | 76 | 79 | 84 | 81 | 85 |
| 40 | 81 | 83 | 84 | 83 | 83 | 80 | 78 | 84 | 79 | 74 | 79 | 74 |
| *Mean* | 81.03 | 81.03 | 81.53 | 80.39 | 81.28 | 80.75 | 81.33 | 80.36 | 79.69 | 77.53 | 79.28 | 77.50 |
| *SD* | 5.15 | 6.24 | 5.69 | 4.85 | 5.43 | 5.44 | 4.76 | 5.60 | 5.51 | 6.45 | 6.04 | 7.40 |
| *Min* | 62 | 56 | 66 | 67 | 65 | 66 | 67 | 62 | 64 | 61 | 60 | 56 |
| *Max* | 88 | 89 | 89 | 86 | 88 | 88 | 89 | 87 | 87 | 88 | 87 | 87 |

*Note*. CTi = competitor-task ineligible; CTe = competitor-task eligible; RA = response alternation; RR = response repetition; PRi = previous response ineligible; PRe = previous response eligible; *SD* = standard deviation.

Table S5

*Number of analysed epochs per participant and condition for the cue-locked EEG data analysis.*

|  | Task Repeat | | | | | | Task Switch | | | | | |
| --- | --- | --- | --- | --- | --- | --- | --- | --- | --- | --- | --- | --- |
|  | CTi | | | CTe | | | CTi | | | CTe | | |
|  | RA | RA | RR | RA | RA | RR | RA | RA | RR | RA | RA | RR |
| ID | PRi | PRe | PRe | PRi | PRe | PRe | PRi | PRe | PRe | PRi | PRe | PRe |
| 2 | 83 | 87 | 84 | 78 | 82 | 87 | 81 | 85 | 82 | 81 | 80 | 79 |
| 3 | 79 | 78 | 83 | 76 | 76 | 74 | 80 | 84 | 74 | 71 | 81 | 73 |
| 4 | 82 | 83 | 80 | 78 | 84 | 76 | 81 | 77 | 80 | 73 | 80 | 81 |
| 5 | 85 | 82 | 85 | 80 | 83 | 85 | 86 | 83 | 80 | 82 | 81 | 82 |
| 6 | 77 | 80 | 77 | 74 | 76 | 76 | 77 | 77 | 77 | 70 | 77 | 69 |
| 7 | 83 | 80 | 82 | 83 | 84 | 84 | 86 | 82 | 81 | 79 | 86 | 84 |
| 9 | 82 | 80 | 82 | 85 | 83 | 87 | 84 | 82 | 78 | 78 | 80 | 85 |
| 10 | 86 | 84 | 84 | 86 | 87 | 82 | 85 | 87 | 85 | 83 | 84 | 83 |
| 11 | 86 | 83 | 86 | 88 | 83 | 86 | 83 | 88 | 88 | 85 | 85 | 88 |
| 12 | 85 | 81 | 81 | 85 | 85 | 84 | 86 | 83 | 78 | 81 | 82 | 83 |
| 13 | 80 | 72 | 77 | 71 | 72 | 69 | 76 | 74 | 77 | 72 | 70 | 71 |
| 14 | 84 | 86 | 84 | 85 | 89 | 86 | 85 | 86 | 78 | 81 | 87 | 83 |
| 15 | 83 | 83 | 82 | 82 | 82 | 82 | 85 | 82 | 86 | 82 | 85 | 80 |
| 16 | 80 | 85 | 84 | 82 | 80 | 75 | 86 | 77 | 84 | 80 | 80 | 80 |
| 17 | 75 | 79 | 81 | 81 | 80 | 82 | 87 | 79 | 83 | 77 | 73 | 76 |
| 18 | 82 | 81 | 83 | 81 | 82 | 85 | 84 | 79 | 77 | 83 | 77 | 83 |
| 19 | 85 | 81 | 83 | 83 | 80 | 83 | 80 | 80 | 77 | 79 | 79 | 81 |
| 20 | 77 | 79 | 68 | 79 | 78 | 74 | 70 | 74 | 70 | 64 | 74 | 71 |
| 21 | 61 | 49 | 67 | 67 | 63 | 64 | 67 | 65 | 64 | 60 | 58 | 55 |
| 23 | 82 | 86 | 83 | 85 | 84 | 86 | 83 | 82 | 83 | 77 | 85 | 82 |
| 25 | 77 | 78 | 76 | 72 | 70 | 71 | 79 | 77 | 71 | 74 | 76 | 70 |
| 26 | 79 | 80 | 77 | 81 | 85 | 76 | 79 | 82 | 78 | 76 | 83 | 74 |
| 27 | 79 | 82 | 80 | 80 | 83 | 79 | 82 | 78 | 84 | 65 | 74 | 74 |
| 28 | 84 | 87 | 85 | 87 | 87 | 86 | 83 | 86 | 86 | 89 | 85 | 84 |
| 29 | 76 | 80 | 83 | 78 | 78 | 75 | 76 | 79 | 79 | 77 | 77 | 81 |
| 30 | 88 | 85 | 87 | 85 | 87 | 85 | 85 | 84 | 84 | 86 | 82 | 84 |
| 31 | 86 | 82 | 80 | 83 | 88 | 76 | 84 | 78 | 81 | 81 | 81 | 80 |
| 32 | 80 | 84 | 80 | 79 | 82 | 77 | 79 | 78 | 76 | 80 | 78 | 77 |
| 33 | 76 | 70 | 76 | 78 | 76 | 76 | 74 | 70 | 68 | 76 | 67 | 73 |
| 34 | 74 | 73 | 71 | 76 | 70 | 72 | 77 | 71 | 74 | 71 | 71 | 71 |
| 35 | 79 | 78 | 68 | 66 | 77 | 73 | 76 | 77 | 70 | 71 | 68 | 64 |
| 36 | 87 | 89 | 88 | 86 | 88 | 85 | 83 | 88 | 88 | 86 | 87 | 87 |
| 37 | 79 | 83 | 83 | 81 | 75 | 82 | 81 | 80 | 81 | 79 | 81 | 74 |
| 38 | 76 | 73 | 76 | 74 | 69 | 71 | 75 | 62 | 70 | 59 | 59 | 54 |
| 39 | 85 | 84 | 85 | 82 | 86 | 83 | 80 | 79 | 75 | 84 | 83 | 85 |
| 40 | 82 | 82 | 84 | 80 | 80 | 80 | 79 | 86 | 81 | 74 | 79 | 78 |
| *Mean* | 80.67 | 80.25 | 80.42 | 79.92 | 80.39 | 79.28 | 80.67 | 79.47 | 78.56 | 76.83 | 78.19 | 77.19 |
| *SD* | 5.03 | 6.84 | 5.29 | 5.30 | 6.10 | 5.98 | 4.68 | 5.94 | 5.78 | 7.13 | 7.07 | 7.99 |
| *Min* | 61 | 49 | 67 | 66 | 63 | 64 | 67 | 62 | 64 | 59 | 58 | 54 |
| *Max* | 88 | 89 | 88 | 88 | 89 | 87 | 87 | 88 | 88 | 89 | 87 | 88 |

*Note*. CTi = competitor-task ineligible; CTe = competitor-task eligible; RA = response alternation; RR = response repetition; PRi = previous response ineligible; PRe = previous response eligible; *SD* = standard deviation. Table S6

*Descriptive statistics for the three dependent variables (RTs, PE, LISAS) across all twelve conditions.*

|  |  |  |  | RTs [ms] | |  | PE [%] | |  | LISAS [a.u.] | |
| --- | --- | --- | --- | --- | --- | --- | --- | --- | --- | --- | --- |
| Task Sequence | CTE | Response Sequence | PRE | *Mean* | *SD* |  | *Mean* | *SD* |  | *Mean* | *SD* |
| Repeat | Ineligible | Alternation | Ineligible | 677 | 124 |  | 1.0 | 1.2 |  | 692 | 130 |
|  |  |  | Eligible | 655 | 111 |  | 0.7 | 1.1 |  | 666 | 114 |
|  |  | Repetition | Eligible | 675 | 117 |  | 1.3 | 1.5 |  | 693 | 120 |
|  | Eligible | Alternation | Ineligible | 680 | 124 |  | 2.1 | 2.2 |  | 712 | 132 |
|  |  |  | Eligible | 680 | 122 |  | 1.7 | 2.4 |  | 703 | 129 |
|  |  | Repetition | Eligible | 673 | 115 |  | 2.6 | 2.5 |  | 715 | 138 |
| Switch | Ineligible | Alternation | Ineligible | 699 | 119 |  | 1.0 | 1.7 |  | 712 | 123 |
|  |  |  | Eligible | 704 | 122 |  | 2.7 | 3.8 |  | 742 | 139 |
|  |  | Repetition | Eligible | 728 | 127 |  | 2.8 | 2.9 |  | 770 | 137 |
|  | Eligible | Alternation | Ineligible | 719 | 133 |  | 5.5 | 4.5 |  | 803 | 155 |
|  |  |  | Eligible | 687 | 118 |  | 2.5 | 3.0 |  | 721 | 124 |
|  |  | Repetition | Eligible | 733 | 122 |  | 5.9 | 5.7 |  | 819 | 141 |

*Note.* CTE = competitor-task eligibility; PRE = previous response eligibility; RTs = response times; PE = percent errors; LISAS = linear integrated speed-accuracy score; a.u. = arbitrary units; *SD* = standard deviation.

**
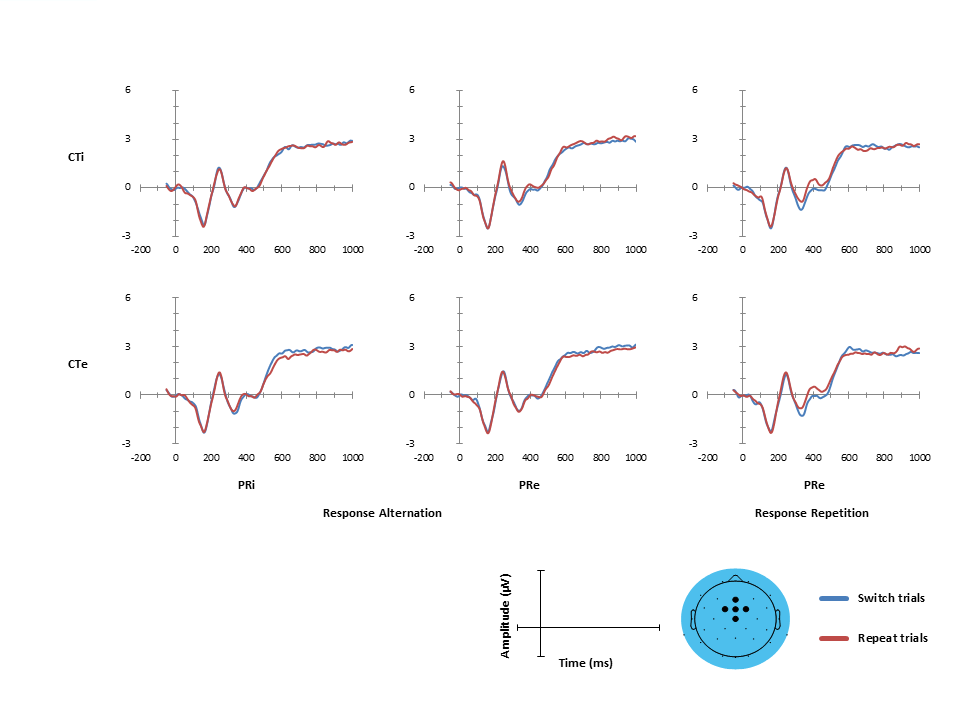
**

**Figure S1** Target-locked original grand-average ERP waveforms, separately for all 12 experimental conditions at fronto-central electrodes as indicated in the scalp map (black dots). Upper panels: CTi, competitor task ineligible. Lower panels: CTe, competitor task eligible. Left-sided panels: PRi, previous response ineligible. Central panels: PRe, previous response eligible, response alternation. Right-sided panels: PRe, previous response eligible, response repetition. Task switch (blue lines) and task repeat (red lines) trials as indicated.


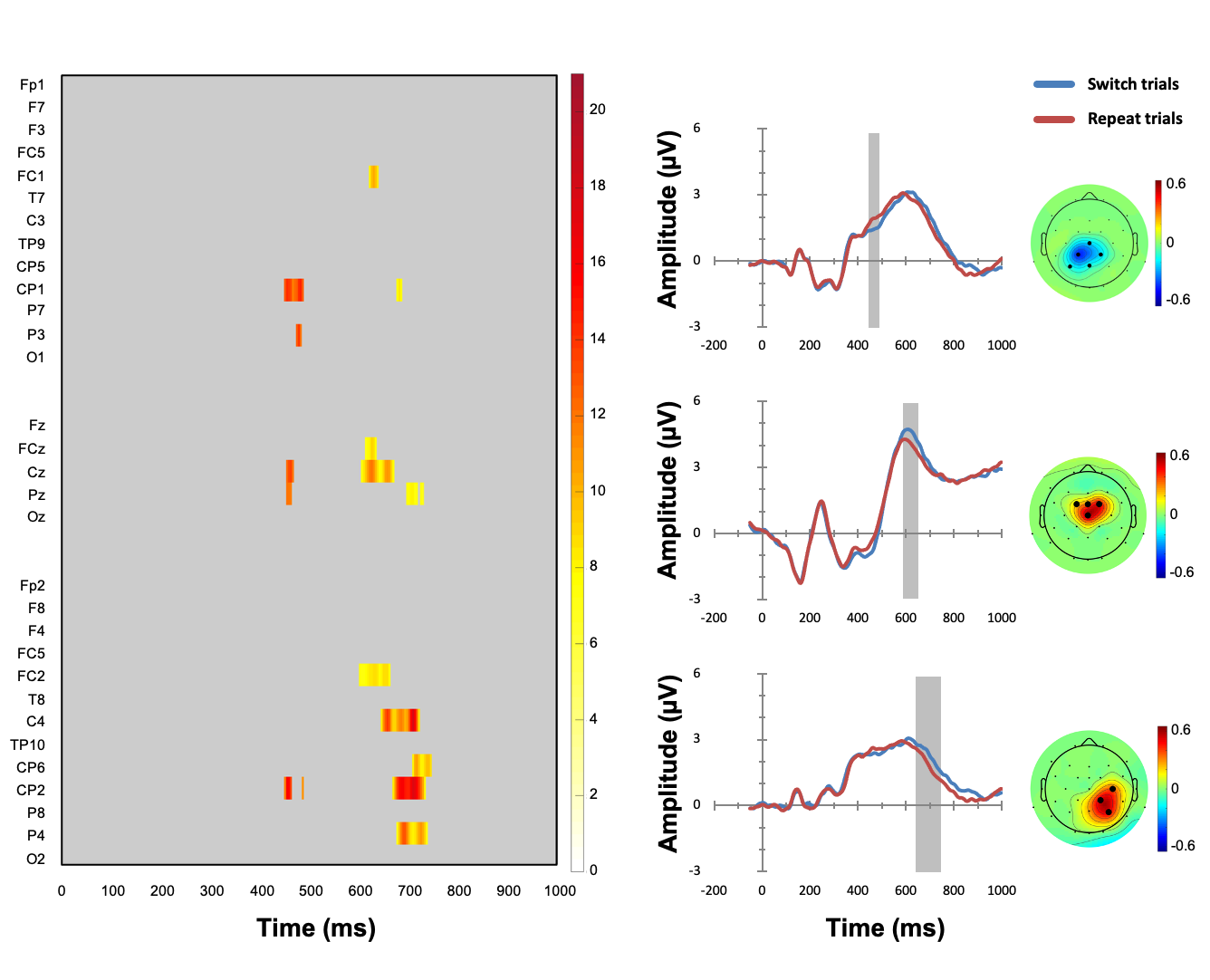


**Figure S2** Results of the repeated-measures TFCE ANOVA including Task Sequence x CTE factors. Left-sided panel: Raster diagram showing time course and topographic distribution of significant Task Sequence main effect (color-coded *F* values). Right-sided panels: Middle panels: Target-locked reconstructed grand-average ERP waveforms, separately for task switch and task repeat trials at the electrodes as indicated in the scalp maps. Right panels: The scalp topography of the switch-related amplitude differences (in μV) in the time windows highlighted with grey shadows in the corresponding ERP waveform.


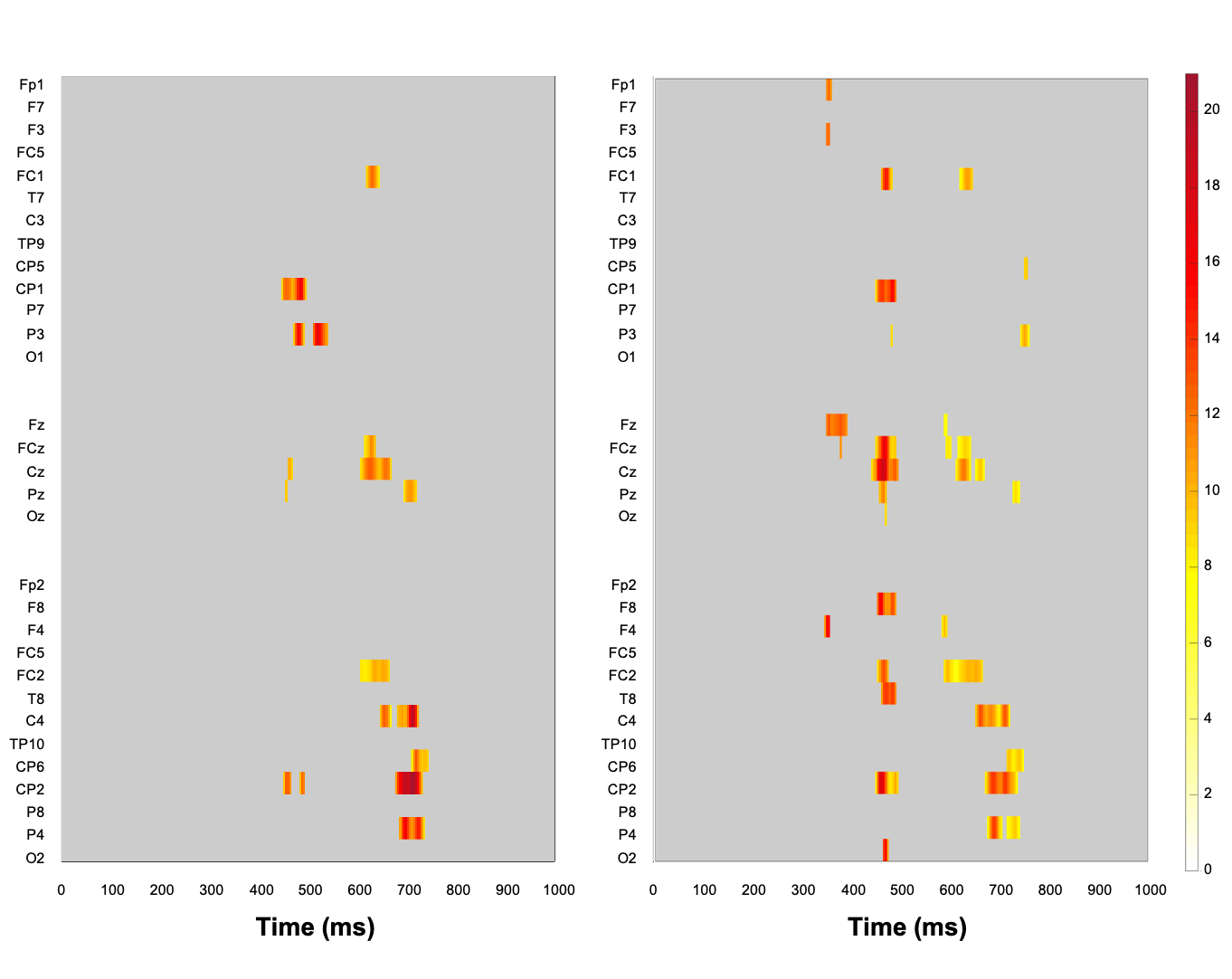


**Figure S3** Results of the repeated-measures TFCE ANOVAs including Task Sequence x PRE (left panel) and Task Sequence x Response Sequence (right panel) factors. Raster diagrams show the time course and topographic distribution of significant Task Sequence main effects (color-coded *F* values).

**
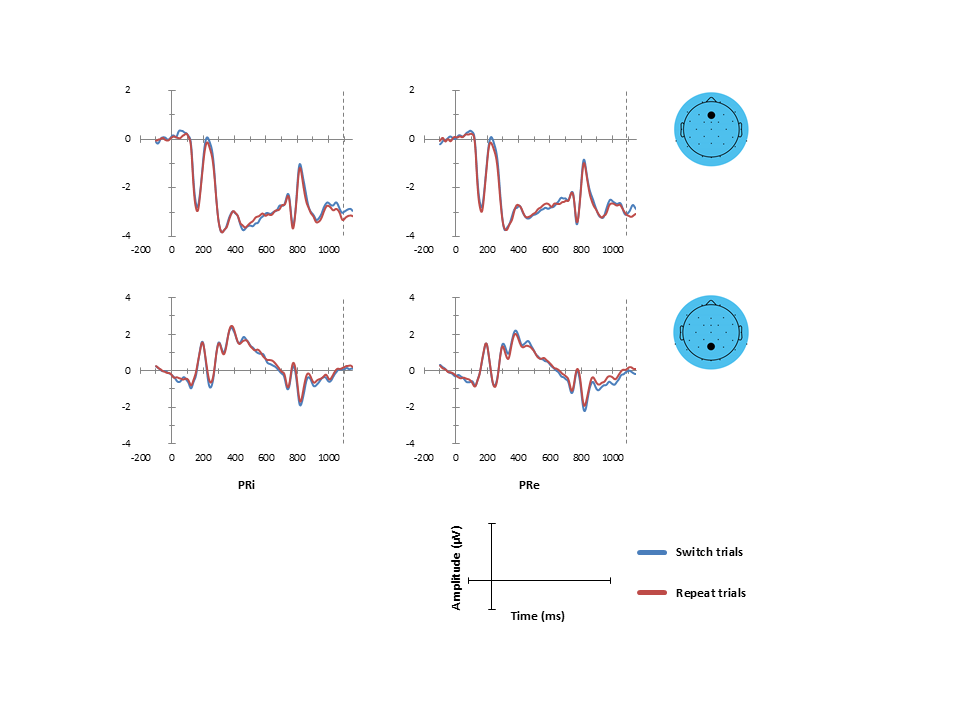
**

**Figure S4** Cue-locked original grand-average ERP waveforms, separately for 4 suitable experimental conditions at electrodes as indicated in the scalp maps (black dots). Left-sided panels: PRi, previous response ineligible. Right-sided panels: PRe, previous response eligible. Task switch (blue lines) and task repeat (red lines) trials as indicated.


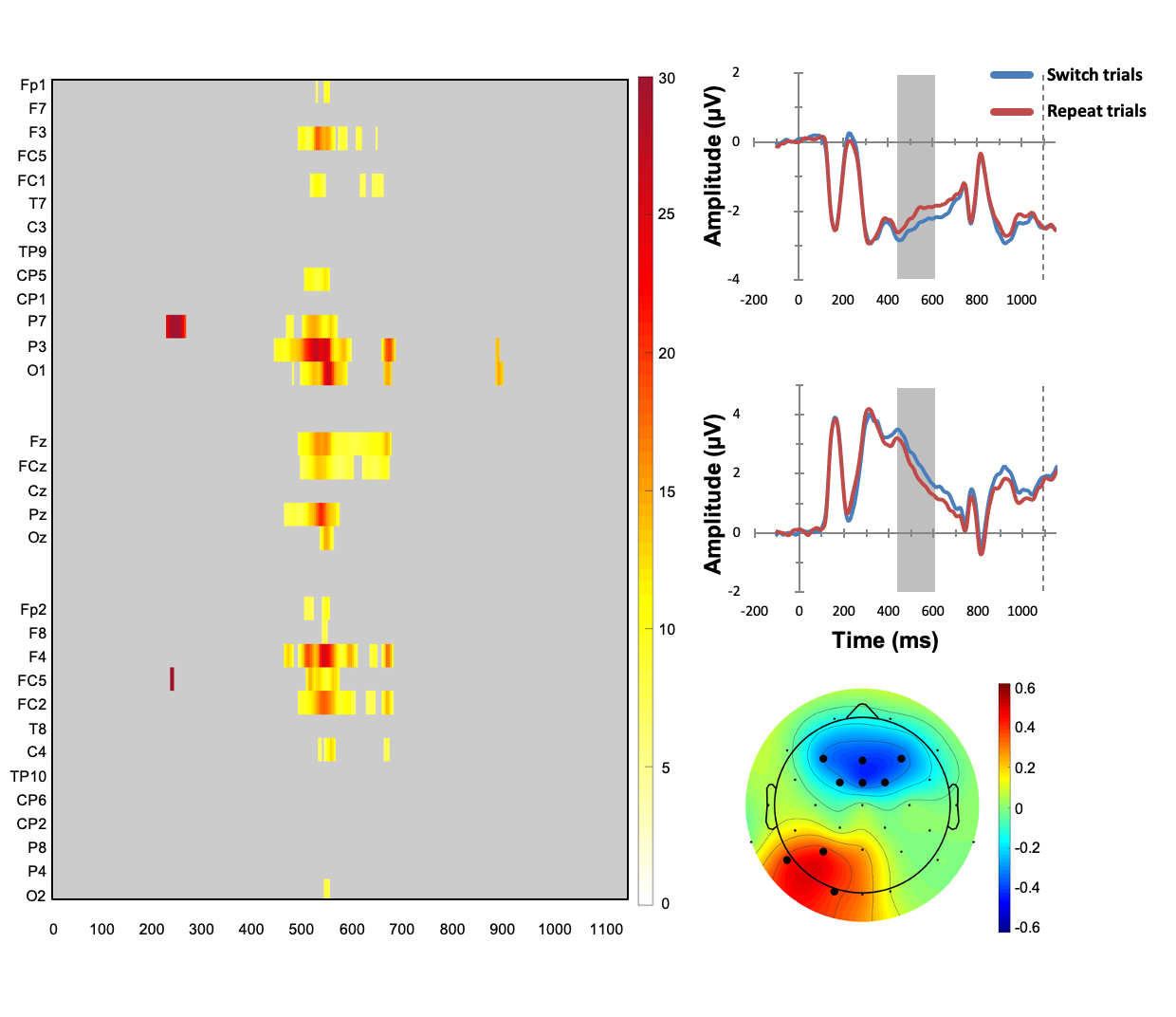


**Figure S5** Left-sided panel: Raster diagram showing time course and topographic distribution of significant main effect for Task Sequence in cue-locked reconstructed grand-average ERP waveforms (color-coded *F* values, alpha-corrected = .025) for one of the two conducted ANOVAs (see Figure 9, for the results of the other ANOVA). Right-sided panels: Upper panel: Cue-locked reconstructed grand-average ERP waveforms, separately for task switch and task repeat trials at frontal and posterior electrodes as indicated in the scalp map below (black dots). Lower panel: The scalp topography of the switch-related amplitude differences (in μV).
